# Genome-centric resolution of novel microbial lineages in an excavated *Centrosaurus* dinosaur fossil bone from the Late Cretaceous of North America

**DOI:** 10.1186/s40793-020-00355-w

**Published:** 2020-03-19

**Authors:** Renxing Liang, Maggie C. Y. Lau, Evan T. Saitta, Zachary K. Garvin, Tullis C. Onstott

**Affiliations:** 1 Department of Geosciences, Princeton University, B88, Guyot Hall, Princeton University, Princeton, NJ 08544 USA; 2 Present address: Institute of Deep-Sea Science and Engineering, Chinese Academy of Sciences, Sanya, China; 3 Integrative Research Center, Section of Earth Sciences, Field Museum of Natural History, Chicago, USA

**Keywords:** *Centrosaurus* fossil bone, Diagenesis, Rare *Actinobacteria*, Halotolerant bacteria, Late Cretaceous, Uncultured microbial lineages

## Abstract

**Background:**

Exceptional preservation of endogenous organics such as collagens and blood vessels has been frequently reported in Mesozoic dinosaur fossils. The persistence of these soft tissues in Mesozoic fossil bones has been challenged because of the susceptibility of proteins to degradation and because bone porosity allows microorganisms to colonize the inner microenvironments through geological time. Although protein lability has been studied extensively, the genomic diversity of microbiomes in dinosaur fossil bones and their potential roles in bone taphonomy remain underexplored. Genome-resolved metagenomics was performed, therefore, on the microbiomes recovered from a Late Cretaceous *Centrosaurus* bone and its encompassing mudstone in order to provide insight into the genomic potential for microbial alteration of fossil bone.

**Results:**

Co-assembly and binning of metagenomic reads resulted in a total of 46 high-quality metagenome-assembled genomes (MAGs) affiliated to six bacterial phyla (*Actinobacteria*, *Proteobacteria*, *Nitrospira*, *Acidobacteria*, *Gemmatimonadetes* and *Chloroflexi*) and 1 archaeal phylum (*Thaumarchaeota*). The majority of the MAGs represented uncultivated, novel microbial lineages from class to species levels based on phylogenetics, phylogenomics and average amino acid identity. Several MAGs from the classes *Nitriliruptoria*, *Deltaproteobacteria* and *Betaproteobacteria* were highly enriched in the bone relative to the adjacent mudstone. Annotation of the MAGs revealed that the distinct putative metabolic functions of different taxonomic groups were linked to carbon, nitrogen, sulfur and iron metabolism. Metaproteomics revealed gene expression from many of the MAGs, but no endogenous collagen peptides were identified in the bone that could have been derived from the dinosaur. Estimated in situ replication rates among the bacterial MAGs suggested that most of the microbial populations in the bone might have been actively growing but at a slow rate.

**Conclusions:**

Our results indicate that excavated dinosaur bones are habitats for microorganisms including novel microbial lineages. The distinctive microhabitats and geochemistry of fossil bone interiors compared to that of the external sediment enrich a microbial biomass comprised of various novel taxa that harbor multiple gene sets related to interconnected biogeochemical processes. Therefore, the presence of these microbiomes in Mesozoic dinosaur fossils urges extra caution to be taken in the science of paleontology when hunting for endogenous biomolecules preserved from deep time.

## Background

Persistence of endogenous organic remains such as DNA and proteins in Mesozoic dinosaurs has long been deemed unlikely due to their lability and gradual breakdown through deep time and diagenesis [1–3]. However, a series of groundbreaking studies has proposed through structural observations, immunohistochemistry, proteomics and in situ microspectroscopic methods [4–11] that endogenous organics such as collagens, blood vessels, erythrocytes and osteocytes appear to be organically preserved in various dinosaur fossils with relatively limited alteration. The reports of collagen peptides from Mesozoic dinosaur fossils are often viewed as particularly exciting in paleontology as they, if genuine, could dramatically enhance our understanding of the evolutionary biology of extinct organisms through deep time [5]. Despite the increasing number of reports about the preservation of endogenous biocomponents in dinosaur fossils over the last decade, their existence remains controversial due to concerns about exogenous contamination from microbial biofilms [12] and other sources associated with analytical procedures [13–15], and to the difficulty in reasonably explaining the mechanisms for their exceptional preservation [5].

Regardless of the authenticity of the preserved endogenous organics in dinosaur fossils, the alternative hypothesis pertaining to microbial biofilms is particularly intriguing from the viewpoint of microbial ecology. Microbial communities from the past and present are well-known to play different roles in biodegradation and biomineralization at various stages throughout the taphonomic history of fossils [16–19]. On the one hand, endogenous organics including soft tissues inside the bone could be rapidly decomposed by intensive metabolic activity of microorganisms after the death of vertebrates [20]. On the other hand, other taphonomic studies have shown that, in some circumstances, microbes might facilitate exceptional preservation of certain soft tissues during post-mortem decay through authigenic mineralization via replacement with phosphates or pyrite [18, 21–23]. The retention of primary soft-tissues within vertebrate bone has been suggested to be enhanced by porosity and permeability reduction from mineral precipitation by microbial biofilms within the bone that are involved in the decomposition of organic matter at early taphonomic stages [23]. It should be noted that the decreasing permeability would not change any inherent thermodynamic instability of these soft tissues, and microscopic cracking can occur in bone apatite as a result of collagen hydrolytic fragmentation, gelatinization, and swelling through hydration [24, 25]. Extreme examples of likely microbially mediated mineralization trapping fossil organics like melanin or steroids, albeit diagenetically altered, are carbonate concretions from the Mazon Creek Formation [26, 27].

The porosity and permeability structure of fossil bone determine the microbial and nutrient exchanges between the bone and the host strata throughout geological time [28]. Therefore, the dynamics of microbial colonization inside the fossil bone would be dependent upon the continuous interaction with the changing hydrogeological environment from post-mortem to recent exposure. Several studies have documented the microbial diversity and activity in relatively recent fossil remains from diverse environments [29–32]. The microbial community profile revealed that most of the DNA obtained from 200 yr to 20 kyr old bones originated from microorganisms that had recently colonized them from the host sediment [29, 30]. Although soft tissues preserved in Mesozoic dinosaur bone have been alternatively interpreted as modern microbial biofilms [12], the microbiome associated with such fossils from deep time remains largely ignored during paleontological and taphonomic studies. In a previous study [28], we analysed freshly-excavated, aseptically-acquired, Late Cretaceous *Centrosaurus* bones and sediment matrix from the Dinosaur Park Formation with a combination of microbiological and chemical techniques. The 16S rRNA gene amplicon survey revealed a diverse microbial community residing within the dinosaur bone. Moreover, several novel microbial lineages were found to be more abundant in the bone than in the surrounding sediment [28].

Due to the limited length of the targeted V4 region of the 16S rRNA gene used in amplicon sequencing [28], the taxonomic status of the dominant novel microbial lineages inside the bone could not be well resolved [28]. Moreover, the molecular survey based on a single biomarker gene precluded us from gaining insight into the potential metabolic functions and their possible ecological roles in biogeochemical cycling and potential taphonomic alteration in fossil bones. To circumvent these limitations, we employed genome-resolved metagenomics with deep sequencing to directly infer the metabolic potential and taxonomic novelty of the predominant microbial populations residing within the *Centrosaurus* bone. By using a co-assembly and consolidated binning strategy [33], we were able to reconstruct 46 metagenome-assembled genomes (MAGs) identified as uncultivated, novel taxonomic lineages varying from class to species level. The enrichment of MAGs from *Nitriliruptoria*, *Deltaproteobacteria* and *Betaproteobacteria* inside the bone suggested that nutrients inside the bone microenvironments facilitated the propagation of these microbial populations during its recent taphonomic history.

## Methods

### Sampling site and fossil bone excavation

A bonebed (BB180) containing *Centrosaurus apertus* (*Ornithischia*; *Ceratopsidae*) in the Late Campanian Dinosaur Park Formation was sampled in 2016 in Dinosaur Provincial Park, Alberta, Canada (50.75 N; 111.4 W). The Dinosaur Park Formation contains alluvial, estuarine, and paralic sedimentary facies deposited during the transgression of the Western Interior Seaway (Bearpaw Sea) in southern Alberta [34]. According to the chronostratigraphic record, the Dinosaur Park Formation interfingers with brackish and marine shales of the overlying Bearpaw Formation [34] and represents one of the units comprising the Judith River Aquifer [35]. The formation of the bone beds appears related to coastal-plain flooding, leading to rapid burial [34]. The Cretaceous strata were subsequently overlain by 1600 to 3800 m of early Tertiary sediments which were later removed by erosion after the Early Eocene [36, 37]. During this interval the strata attained maximum burial temperatures of 90–120 °C, and δ^18^O analyses of diagenetic minerals suggested that the formation water was always dominated by fresh meteoric water, especially during post-Early Eocene erosion [36]. Glacial ice sheets up to 2 km thick covered the strata during the Pleistocene [37] during which the strata were recharged with fresh, Na-HCO_3_-Cl water like that of the Milk River aquifer immediately to the south [37, 38]. Nonetheless the hydrology of these strata is complicated leading to enormous salinity gradients, and the sampling site is located < 10 km from a well where a Na-Cl formation water salinity exceeds 15,000 ppm [37] and this salinity likely originates from the bounding marine shales [39].

The detailed aseptic sampling and transport procedures and the permit documents for collecting dinosaur bones were previously described [28]. Briefly, the *Centrosaurus* bones were first partially exposed at one end from a vadose zone outcrop after removing the sandstone and mudstone overburden from the bone-bearing horizon. In order to minimize contamination, a small, partial *Centrosaurus* rib sample, representing the unexposed end, and the surrounding adjacent mudstone were collected together as a whole using sterilized equipment. The collected bone samples were immediately stored on ice in a cooler before transporting to the camp freezer [28]. All samples were shipped to Princeton University on blue ice packs and stored at –80 °C prior to DNA extraction and other analyses.

### Bone pretreatment and geochemical characterization

The mudstone encapsulated bone was processed inside a UV sterilized laminar flow hood. The fossil bone fragments and adjacent mudstone were carefully separated and bone surface was scraped off (hereafter referred to as scrapings) with a flame-sterilized autoclaved razor. The cleaned bone with outer surface removed, along with the scrapings and the surrounding mudstone matrix were powdered separately with sterilized mortars and pestles. To determine the water-extractable anions, 0.1 g of each powder fraction was thoroughly mixed with 1 mL Milli-Q H_2_O and incubated overnight before measurement. The slurry was centrifuged at 14,000×g for 5 min and the supernatant was then filtered with 0.22 μm filter membrane for ion chromatography analyses. All samples (in triplicate) were analyzed on a Dionex chromatography system equipped with an IC25 Ion Chromatograph, AS40 autosampler, a LC25 chromatography oven and an EG40 eluent generator. Anions were separated using a Dionex IonPac AS15 (3 × 150 mm) analytical column connected to a Dionex IonPac AG15 (3 × 50 mm) guard column. The oven was maintained at 30 °C and the flow rate was set at 0.35 mL/min. The KOH gradient generated by the EG40 eluent generator was as follows: 0–10 min, 5 mM; 10–16 min, gradient from 5 to 40 mM; 16–30 min, isocratic at 40 mM; 30–33, 40 mM to 5 mM; and a final isocratic run (33–40 min) at 5 mM.

### DNA extraction and metagenomic sequencing

The DNA used in this study was the same material recovered from the bone, adjacent mudstone and scrapings as described in our previously publication [28]. Briefly, DNA was extracted from powdered bone (5 g), a slurry of EDTA demineralized bone (5 g), and mudstone (10 g) using DNeasy PowerMax Soil Kit (QIAGEN, Germany) according to the manufacturer’s instruction. The EDTA demineralized bone was incorporated for DNA extraction because pre-treatment with EDTA has been frequently used to remove the mineral phase to recover collagen peptides from dinosaur bones in previous studies [6–8, 40]. A negative control with bone- and mudstone-free reagents were incorporated to monitor contaminant DNA that could be introduced during DNA extraction from the reagents and laboratory environments. The DNA yield in the blank control was found to be below detection (< 0.01 ng/μL) [28] and thus was not included for subsequent metagenomic sequencing.. The library preparation for the resultant DNA from each sample was performed using Nextera DNA Library Prep kit (Illumina, San Diego, CA, USA). The library from each sample with unique barcode were pooled and then sequenced on an Illumina Hiseq 2500 (150-bp, paired-end) at the Genomics Core Facility, Princeton University. Three metagenomes from the bone powder (1B5g), EDTA-treated bone powder (1BEDTA) and adjacent mudstone (1M10g) were generated with a total of ~ 120 million paired-end reads of 2 × 150 bp.

### Contigs assembly and reconstruction of MAGs

The raw sequences were quality-filtered using the Trim Galore pipeline in the Galaxy server at Princeton University (http://galaxy.princeton.edu) as described previously [41]. The clean reads from the three metagenomes were co-assembled with MEGAHIT v1.1.4 [42] using paired-end mode with the settings of *k*-min = 27, *k*-max = 137, *k-*step = 10. The co-assembled contigs (> 1.5 kb) were binned with three different tools, namely MetaBAT v 2.12.1, [43], MaxBin v2.0 [44] and CONCOCT v1.1.0 [45] using the default settings in the “Binning module” implemented in MetaWRAP v0.8 [33]. The MAGs generated with the above three different algorithms were consolidated with the “Bin_refinement module” in MetaWRAP v0.8 [33] using a minimum cutoff value of 80% for completeness and a maximum cutoff value of 10% for contamination as assessed by CheckM v1.0.11 [46]. The consolidated MAGs were further re-assembled with the “Reassemble_bins module” in MetaWRAP v0.8 [33]. Briefly, all metagenomics reads were mapped back to each MAG with “strict” (exact match) and “permissive” (allowing 3 mismatches) options. The mapped reads from each MAG were then reassembled using SPAdes v3.13.0 [47] and a set of k-mer sizes (21, 33, 55, 77) in MetaWRAP v0.8 [33]. The quality of the reassembled MAGs were assessed with CheckM v1.0.11 [46] and those MAGs with a minimum completeness of 90% and a maximum contamination of 10% were retained for downstream analyses.

### Metabolic annotation and phylogenetic analyses

The functional genes (protein coding sequences, CDS) from all MAGs were predicted and annotated using Prokka v1.13 [48] and DFAST tools [49] against TIGRFAM and COG databases. The identified functional genes encoding enzymes of interest, such as microbial collagenase, alkane 1-monooxygenase and naphthalene 1,2-dioxygenase were confirmed by blastp against NCBI nr database when needed. The metabolic pathways for major nutrients metabolism such as carbon and nitrogen were predicted using the automated annotation server RAST (Rapid Annotation using Subsystem Technology) [50] and the KEGG BlastKOALA tool [51] with the default settings.

The 16S rRNA gene in the MAGs was retrieved using the commands (anvi-script-FASTA-to-contigs-db GENOME.fa and anvi-get-sequences-for-hmm-hits -c GENOME.db --hmm-source Ribosomal_RNAs) implemented in Anvi’o 5.2 [52] and RNAmmer 1.2 [53]. The closely related 16S rRNA gene sequences (top 3 hits) from both cultured and uncultured microorganisms were retrieved from the NCBI GenBank database. All 16S rRNA gene sequences were aligned with MUSCLE v3.8.31 [54] and the phylogenetic tree was constructed using the Maximum Likelihood method based on the Tamura-Nei model using MEGA v7.0.20 [55]. Phylogeny of the MAGs from this study and their close relatives (MAGs or genomes of cultivated organisms from NCBI database, accessed in April, 2019) was also assessed based on the same concatenated 16 single-copy ribosomal proteins that had been used to construct the tree of life [56]. The sequences of the ribosomal proteins were extracted from all MAGs and further concatenated for alignment with MUSCLE v3.8.31 in Anvi’o 5.2 [52]. The alignment of the concatenated amino acid sequences was trimmed using trimAl v.1.2 [57] and then the phylogenetic tree was constructed with RAxML v. 8.1.17 [58] using the PROTGAMMAILGF model for amino acid sequence evolution and 1000 bootstraps. The newick tree was viewed and refined using the online iTOL software [59].

### Other genome-centric analyses

The taxonomic classification of all MAGs was also performed using Genome Taxonomy Database Toolkit (GTDB-Tk v 0.3.0) [60] to support the novelty of these microbial lineages. The phylogenetic placement in GTDB-Tk was based on multiple sequence alignment of 120 bacterial and 122 archaeal marker genes from MAGs and the most comprehensive genomes collection in the database (release R04-RS89, June 19th, 2019). Due to the taxonomic novelty of most MAGs, average amino acid identity (AAI) between particular MAGs and other publicly available genomic relatives was calculated using the command (aai-matrix.bash) implemented in the Enveomics toolbox [61]. The relative abundance of all MAGs across the bone, EDTA-treated bone and adjacent mudstone was calculated using the “Quant_bin” module in MetaWRAP v0.8 [33]. According to the taxonomic assignment of each MAG, the microbial community was also constructed by grouping all MAGs into their affiliated taxa at various taxonomic levels. In order to further characterize gene functions in the dominant MAGs in the bone, the gene clusters that are different from genomes from other environments were identified and visualized using the pangenomics workflow in Anvi’o v5.2 [52] with the following parameters (anvi-pan-genome –g Euzebya-GENOMES.db \ --project-name “Euzebya_Pan_new” \ --output-dir Euzebya \ --num-threads 12 \ --minbit 0.5 \--mcl-inflation 2) Lastly, the Growth Rate Index (GRiD) was calculated from all bacterial MAGs to infer in situ growth rates of microbial populations in the bone and adjacent mudstone based on the ratio of coverage at the peak (origin of replication, *ori*) and trough (terminus, *ter*) regions as determined by mapping the metagenomic reads to each MAG [62]. The validity of GRiD values was further tested using the coverage information of chromosome initiator replication gene (*dnaA*) and deletion-induced filamentation (*dif*) sequences across the genome [62]. The GRiD values from the MAGs are considered valid for downstream analyses only if the *dnaA/ori* and *ter/dif* coverage ratios are above 0.8 and the species heterogeneity is low (< 0.3).

### Re-analyses of 16S rRNA gene amplicon data

Although a preliminary analysis of 16S rRNA gene amplicon data has been reported [28], a more in-depth examination of the same dataset was performed to support the genome-centric analyses in this work. The relative abundance of predominant microbial lineages in the dinosaur bone and adjacent mudstone replicates were calculated for pair-wise comparison at various taxonomic levels depending on the resolution of 16S rRNA gene amplicon approach. *P-values* were derived from Student’s t-test to determine whether there were any significant differences in the major taxonomic groups between the bone and adjacent mudstone. The OTU Table (11,467 OTUs across 8 samples) previously generated [28] and all available geochemical parameters were used to perform Canonical Correspondence Analysis (CCA) using the vegan and phyloseq packages [63] in R software v3.5.1. Additionally, the relative abundance of the dominant taxa at the class level was incorporated in the CCA plot to examine their correlation with the geochemical data. The distance matrix for the ordination was based on Bray-Curtis distances and the explanatory variables were chosen by stepwise model selection with permutation tests.

### Metaproteomic analyses

Due to the inherent challenges associated with protein extraction from fossil bones with low biomass, four different approaches were used to extract proteins from the fossil bone and adjacent mudstone. In the first protocol fractions containing proteins (precipitates collected from step 7 and 10 of the DNA extraction procedures described above) from the mudstone and EDTA-treated bone were subject to protein purification using a standard methanol/acetone protocol as described previously [64]. The second protocol was adapted from an earlier study [40] that reported the successful identification of collagen peptides from fossil specimens of *Brachylophosaurus Canadensis*. Briefly, direct protein extraction was attempted by incubating the bone and mudstone powder (1 g each) with 6 M guanidine-HCl at 65 °C overnight to dissolve any potential endogenous collagen peptides; In the third protocol the proteins were extracted using a previously described method optimized for soil metaproteomics [65]. Bone and mudstone powder (5 g of each) were thoroughly mixed with 10 mL alkaline SDS (sodium dodecyl sulphate) buffer and then incubated in water bath (100 °C) for 10 mins. The protein was recovered by trichloroacetic acid precipitation and further cleaned following acetone wash as previously described [65]. The fourth protocol utilized a commercial kit (NoviPure Soil Protein Extraction Kit, QIAGEN, Germany) to extract proteins from the bone (3 g) and mudstone powder (5 g) following the manufacture’s procedures.

The protein pellets from each extraction method were dissolved in 6 M guanidine-HCl and sonicated 5 times for 30 s with 1 min rest on ice in between each cycle. Tris(2- carboxyethyl)phosphine (TCEP) was added to 5 mM final concentration and incubated at 60 °C for 10 min. Chloroacetamide was added (15 mM) and then incubated in the dark at room temperature for further 30 min. Samples were diluted 1:10 with digestion buffer (10% Acetonitrile, 25 mM Trish pH 8.5) and 2 μg of Trypsin Gold (Promega) was added to each sample and incubated end-over-end at 37 °C for 16 h. Samples were acidified by adding trifluoroacetic acid (0.2% final concentration) and were further desalted using Stage Tips [66]. Samples were dried completely in a SpeedVac and resuspended with 20 μL of 0.1% formic acid (pH 3). A 5 μL aliquot of the sample was injected per run using an Easy-nLC 1200 UPLC system. Samples were loaded directly onto a 45 cm long 75 μm inner diameter nano capillary column packed with 1.9 μm C18-AQ (Dr. Maisch, Germany) mated to metal emitter in-line with an Orbitrap Fusion Lumos (Thermo Scientific, USA). The mass spectrometer was operated in data dependent mode with the 120,000 resolution MS1 scan (AGC 4e5, Max IT 50 ms, 400–1500 m/z) in the Orbitrap followed by up to 20 MS/MS scans with CID fragmentation in the ion trap. Dynamic exclusion list was invoked to exclude previously sequenced peptides for 60s if sequenced within the last 30s and maximum cycle time of 3 s was used. Ion-trap was operated in Rapid mode with AGC target 2e3, maximum IT of 300 ms and minimum of 5000 ions.

Protein identification from all metaproteomes was performed against various databases with the open-source software MaxQuant (v. 1.6.0.1) [67]. To identify collagen peptides potentially preserved in the fossil bone, a database was built using all protein sequences from the Uniprot Vertebrates database and collagen peptides reported from Mesozoic dinosaur fossils [8, 10]. The proteome (all CDSs) from all MAGs were predicted by Prodigal [68] and then merged to create another database for identifying expressed proteins from the microbial populations residing in the fossil bone. The cleavage enzyme Trypsin/P was selected and two missed cleavages were allowed. Contaminant sequences such as human keratins, chicken collagens, and bovine serum albumin were automatically included during the search with MaxQuant v1.6.0.1 [67]. The maximum false-discovery rate was set to 0.05 for both the peptide-spectrum matches (PSMs) and proteins using the target-decoy strategy. The validity of identified proteins was based on the criterion of at least one unique peptide per protein.

## Results and discussion

### Geochemical characteristics and correlation to microbial community

The pH of the water extract from the bone was 5 whereas that of the adjacent mudstone was 6.5. The slightly acidic microenvironments inside the *Centrosaurus* bone might facilitate dissolution of hydroxyapatite and contribute to the observed higher concentration of water soluble PO_4_^3−^ in the bone relative to the mudstone (Additional file 2: Table S1). The concentration of SO_4_^2−^ and NO_3_^−^ was much higher than other anions (F^−^, Cl^−^, PO_4_^3−^, NO_2_^−^) in both the bone and the mudstone (Additional file 2: Table S1). Surprisingly, the SO_4_^2−^ and NO_3_^−^ concentrations in the bone were 10 times (4910 μg/g) and two times (1210 μg/g) higher, respectively, than those in the adjacent mudstone suggesting a more oxidizing microenvironment. Formate, acetate, lactate and proprionate were below detection limits (1.3 μg/g) in both the bone and the mudstone. The CCA analysis revealed that pH, SO_4_^2−^ and NO_3_^−^ were more important in influencing the microbial community in the bone relative to other geochemical parameters (Additional file 1: Fig. S1). Our previous 16S rRNA gene amplicon data revealed that the classes *Nitriliruptoria*, *Acidimicrobiia*, *Betaproteobacteria* and *Deltaproteobacteria* were more abundant in the *Centrosaurus* bone relative to the mudstone [28]. The statistically significant difference between bone and adjacent mudstone was supported by performing pair-wise comparison of the dominant groups (Additional file 2: Fig. S2). The CCA analysis suggests that these dominant classes in the bone were positively correlated with SO_4_^2−^ and NO_3_^−^ (Additional file 1: Fig. S1). In contrast, the most abundant *Actinobacteria* in the mudstone was negatively correlated with SO_4_^2−^ and NO_3_^−^ (Additional file 1: Fig. S1)_,_ suggesting that these common soil *Actinobacteria* lineages prefer to reside in less oxidized environments.

### Reconstruction of MAGs representing phylogenetically novel lineages

A total of 180,537 contigs (≥1 kb) were co-assembled from three metagenomes (1B5g, 1BEDTA and 1M10g) with a combined input of ~ 120 million quality-filtered reads (paired-end, 150-bp). By integrating 3 different algorithms and a consolidation strategy [33], we were able to recover 59 MAGs (> 80% complete and < 10% contamination) from the co-assembled contigs. Further re-assembly resulted in 46 MAGs (90% complete) that were selected for downstream analyses (Additional file 3:Table S2). According to the standards proposed by Bowers et al. [69], all MAGs qualified as high-quality draft genomes (> 90% complete and < 5% contamination) except that the contamination level of three MAGs (Dino_bin7, 6.15%; Dino_bin26, 6.36%; and Dino_bin50, 5.13%) was slightly above 5% (Additional file 3: Table S2).

**Fig. 1:**
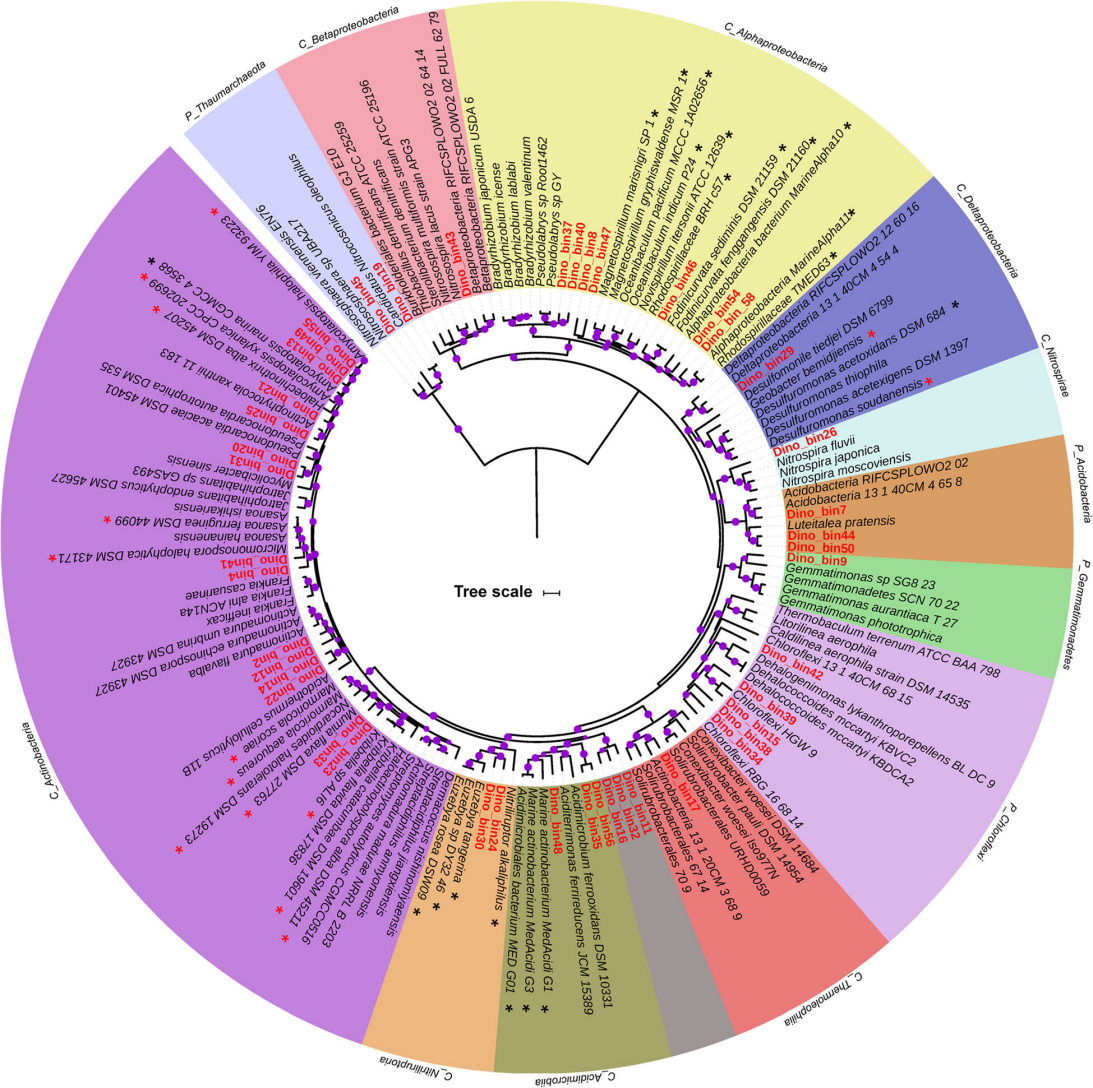
Phylogenetic tree of MAGs and their close genomic representatives from diverse environments. The Maximum-likelihood phylogenomic tree was based on 16 concatenated ribosomal proteins. The black and red asterisks indicate those microorganisms originating from marine environments and hypersaline environments such as salt lakes and subsurface brines, respectively. The purple dots represent bootstrap values > 70% (bootstrap values were generated from 1000 replications) whereas the bootstrap value of those nodes without a dot was less than 70%. Note: The taxa starting with “P” and “C” refer to their taxonomic level of phylum and class, respectively. The scale bar corresponds to 0.1 substitutions per amino acid position

**Fig. 2:**
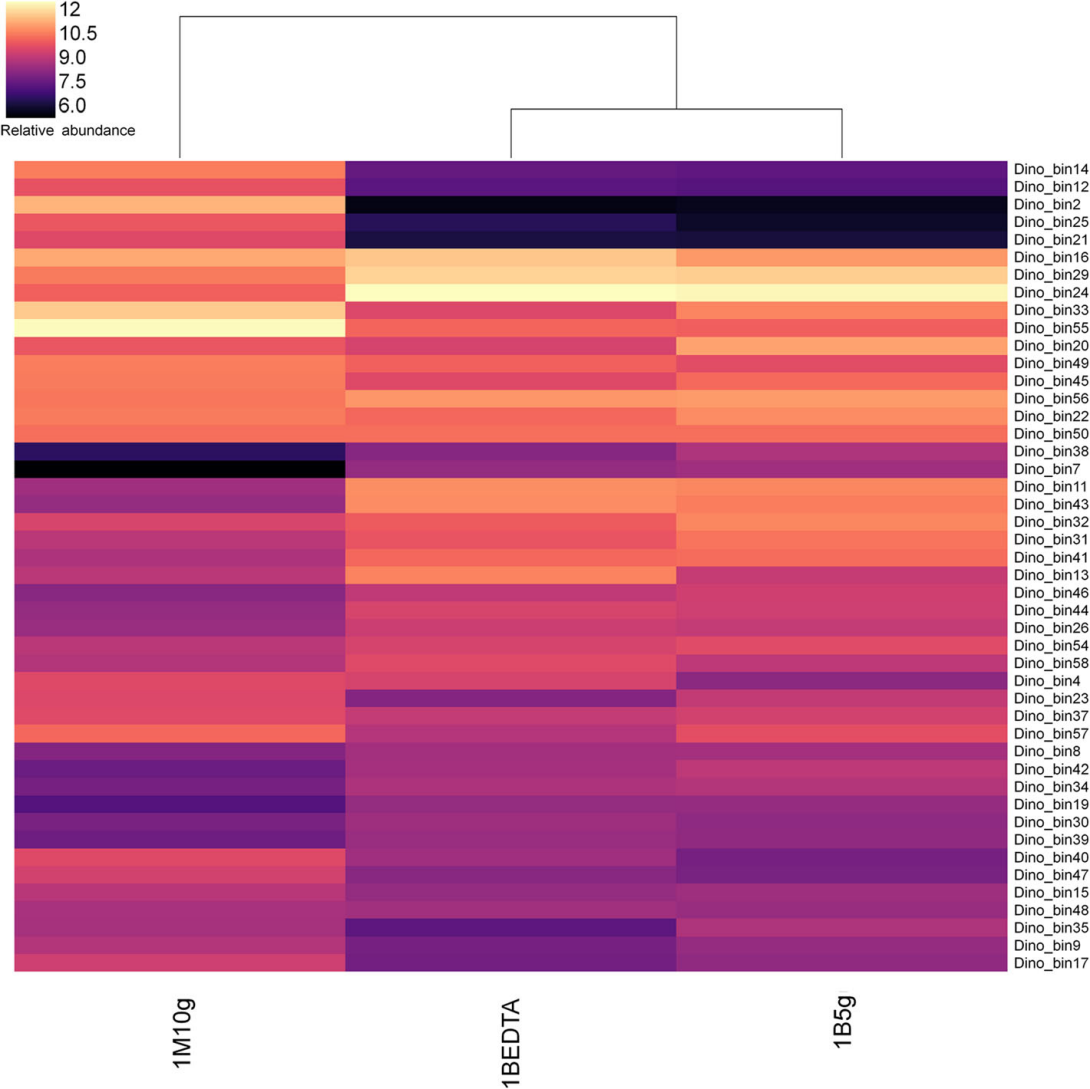
Relative abundance of MAGs across the three metagenomes (1B5g, 1BEDTA and 1M10g). The abundance of each MAG was calculated from the reads mapped in the metagenome and normalized to the individual sample size as genome copies per million reads

According to phylogenomic analyses (Fig. 1) and genome-based taxonomy (GTDB-Tk) (Additional file 4: Table S3), the taxonomic annotation revealed that these 46 high-quality MAGs represent 6 bacterial phyla (*Actinobacteria*, *Proteobacteria*, *Nitrospira*, *Acidobacteria*, *Gemmatimonadetes* and *Chloroflexi*) and 1 archaeal phylum (*Thaumarchaeota*). No MAGs or contigs belonging to Eukaryotes, such as Fungi, were detected. The clustering of 1B5g and 1BEDTA relative to 1M10g based upon the relative abundance of the MAGs further confirmed the distinct microbial community structure of the bone versus that of the mudstone [28] (Fig. 2). The MAGs associated with *Delatproteobacteria* (Dino_bin29), *Nitriliruptoria* (Dino_bin24) and *Betaproteobacteria* (Dino_bin43) were more abundant (4–13 times) inside the bone than the mudstone, whereas many *Actinobacteria* MAGs (Dino_bin2, Dino_bin12 and Dino_bin14, Dino_bin25 and Dino_bin55) were more abundant in the mudstone than the bone (Fig. 2 and Additional file 5: Table S4). Overall, the MAGs of the bone microbial community represented the same dominant microbial lineages (Fig. 2 and Additional file 1: Fig. S2) as previously determined by 16S rRNA gene amplicon sequencing with *Nitriliruptoria* (Dino_bin24) comprising 26–28% of the *Centrosaurus* bone microbiome [28].

**Fig. 3:**
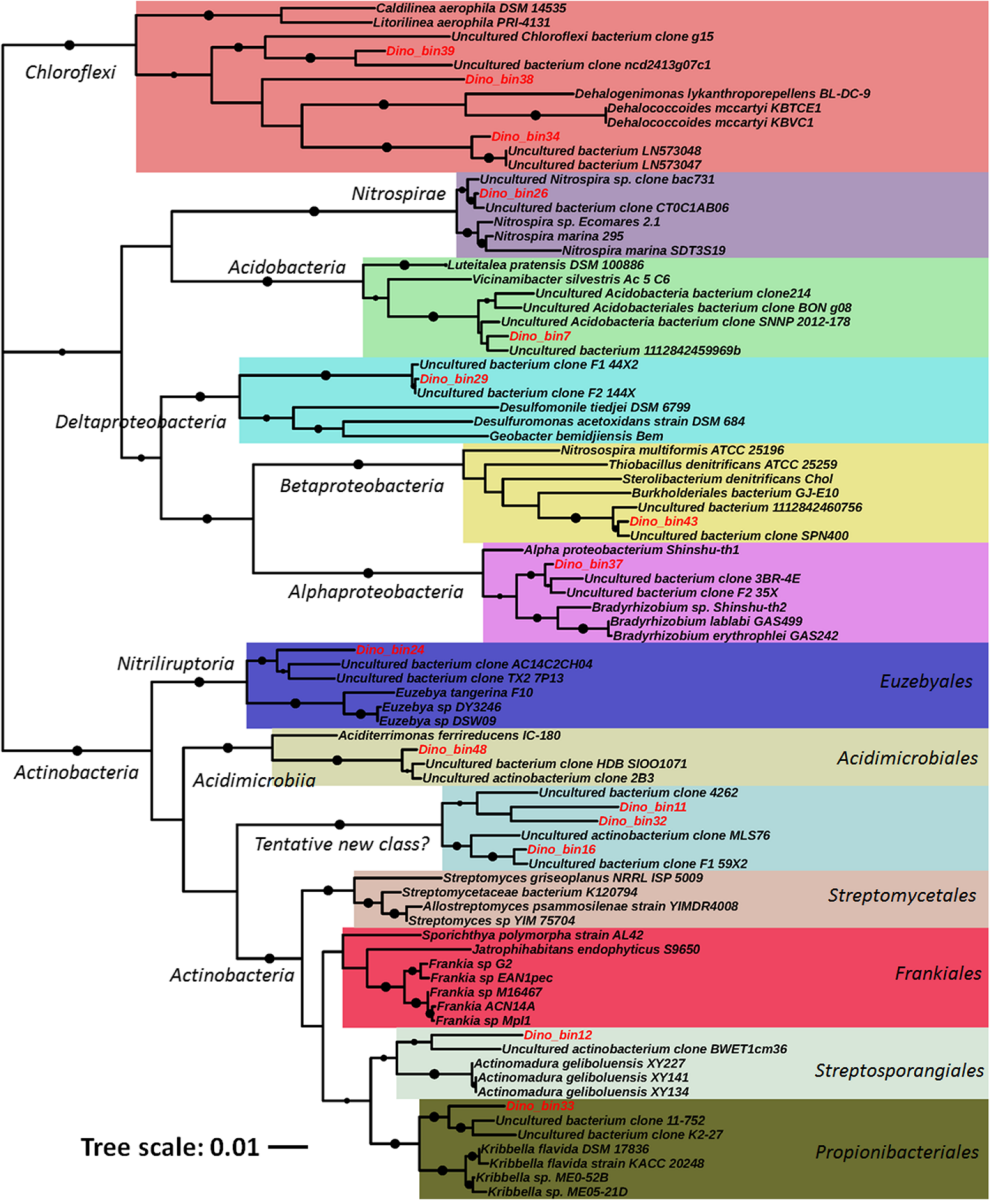
Phylogenetic tree based on 16S rRNA genes from MAGs and close relatives of culture and uncultured organisms. The black dots represent bootstrap values > 70% (bootstrap values were generated from 1000 replications). The 16S rRNA genes from MAGs in this study were highlighted in bold and red. The scale bar corresponds to 0.01substitutions per nucleotide position

The phylogenomic tree revealed that many of the MAGs were distantly related to the publicly available genomes as evidenced by their formation of separate clades (Fig. 1). Such observations underline the phylogenetic novelty of microbial populations from the *Centrosaurus* fossil bone. Notably, the *Actinobacteria* MAGs (Dino_bin11, Dino_bin16 and Dino_bin32) are placed within a novel clade with no previously reported neighboring genomic relatives and thus might represent a novel class or order within the phylum *Actinobacteria*. Partial to nearly full-length 16S rRNA genes were recovered from 15 of the MAGs (Additional file 6: Table S5) to construct a phylogenetic tree with their close relatives of uncultivated and cultivated organisms (Fig. 3). The 16S rRNA genes from the 15 MAGs closely clustered with environmental sequences from uncultivated bacteria as separate clades (Fig. 3). All 15 MAGs except for the *Nitrospira*-like organism (Dino_bin26, ~ 98% identical to *Nitrospira marina* Nb-295) were tentatively identified as novel lineages at least at the species level based on the low homology (84–95%) with known cultivated microbes (Additional file 6: Table S5). Among them, the MAGs affiliated with *Deltaproteobacteria* (Dino_bin29), *Chloroflexi* (Dino_bin34, Dino_bin38, and Dino_bin39) and *Actinobacteria* (Dino_bin11, Dino_bin16 and Dino_bin32) might represent novel classes or orders in light of the low similarity (84–87%) of their 16S rRNA gene to that of known microbes (Additional file 6: Table S5). The taxonomic classifications assigned by the GTDB-Tk tool [60] confirmed that the abovementioned 7 MAGs can only be identified to class or order level from genomic comparison to the genome collection in the latest database (Additional file 4: Table S3). The taxonomic novelty of these MAGs was further supported by the extremely low AAI values (Additional file 1: Fig. S3-S7) based on the previously proposed thresholds for species (95%) and genus (65%) [70]. For the MAGs affiliated with *Chloroflexi* (Dino_bin42), *Deltaproteobacteria* (Dino_bin29) and *Actinobacteria* (Dino_bin11, Dino_bin16 and Dino_bin32), the maximum AAI value was generally below ~ 50% when compared with the closely related genomic representatives (Additional file 1: Fig. S5-S7). Therefore, the majority of the reconstructed MAGs belong to completely new microbial lineages at least at species level based on 16S rRNA phylogeny, phylogenomics, AAI profiling and genome-based taxonomy.

### Prevalence of halotolerant organisms in the *Centrosaurus* fossil bone

Several of the dominant lineages including *Euzebyales* (Dino_bin24 and Dino_30), *Acidimicrobiia* (Dino_bin48) and most of the *Alphaproteobacteria* MAGs (Dino_bin46, Dino_bin54 and Dino_bin58) were phylogenetically related to microorganisms originating from marine environments (Fig. 1). The two predominant *Euzebyales*-related MAGs (Dino_bin24 and 30) in the bone were closely related to *Actinobacteria* (*Euzebya spp*.) isolated from various marine environments in Japan and China [71–73]. Pangenomics analysis of the *Euzebya-related* MAGs revealed that three gene clusters were present in the terrestrial MAGs from the *Centrosaurus* bone but absent from the marine counterparts (Additional file 1: Fig. S8). Additionally, the *Acidimicrobiia*-related MAG (Dino_bin48) and *Alphaproteobacteria* MAGs (Dino_bin46, Dino_bin54 and Dino_bin58) were most phylogenetically related to draft genomes recovered from the Mediterranean deep chlorophyll maximum [74] and Tara Oceans metagenomic datasets [75, 76], respectively. Moreover, the *Nitrospira*-related MAG (Dino_bin_26) showed the highest similarity (~ 98%) to marine nitrite-oxidizers such as *Nitrospira marina* [77] and *Nitrospira marina* Ecomares 2.1 [78] based on 16S rRNA phylogeny (Fig. 3 and Table S4). Apart from microorganisms from marine environments, the *Deltaproteobacteria* MAG (Dino_bin29) and many *Actionbacteria* MAGs (Dino_bin23, Dino_bin33, Dino_bin4, Dino_bin41, Dino_bin13, Dino_bin49, Dino_bin55) were closely related to microorganisms found in other high salinity environments such as salt lakes, mangrove soil and subsurface brine (Fig. 1).

**Fig. 4:**
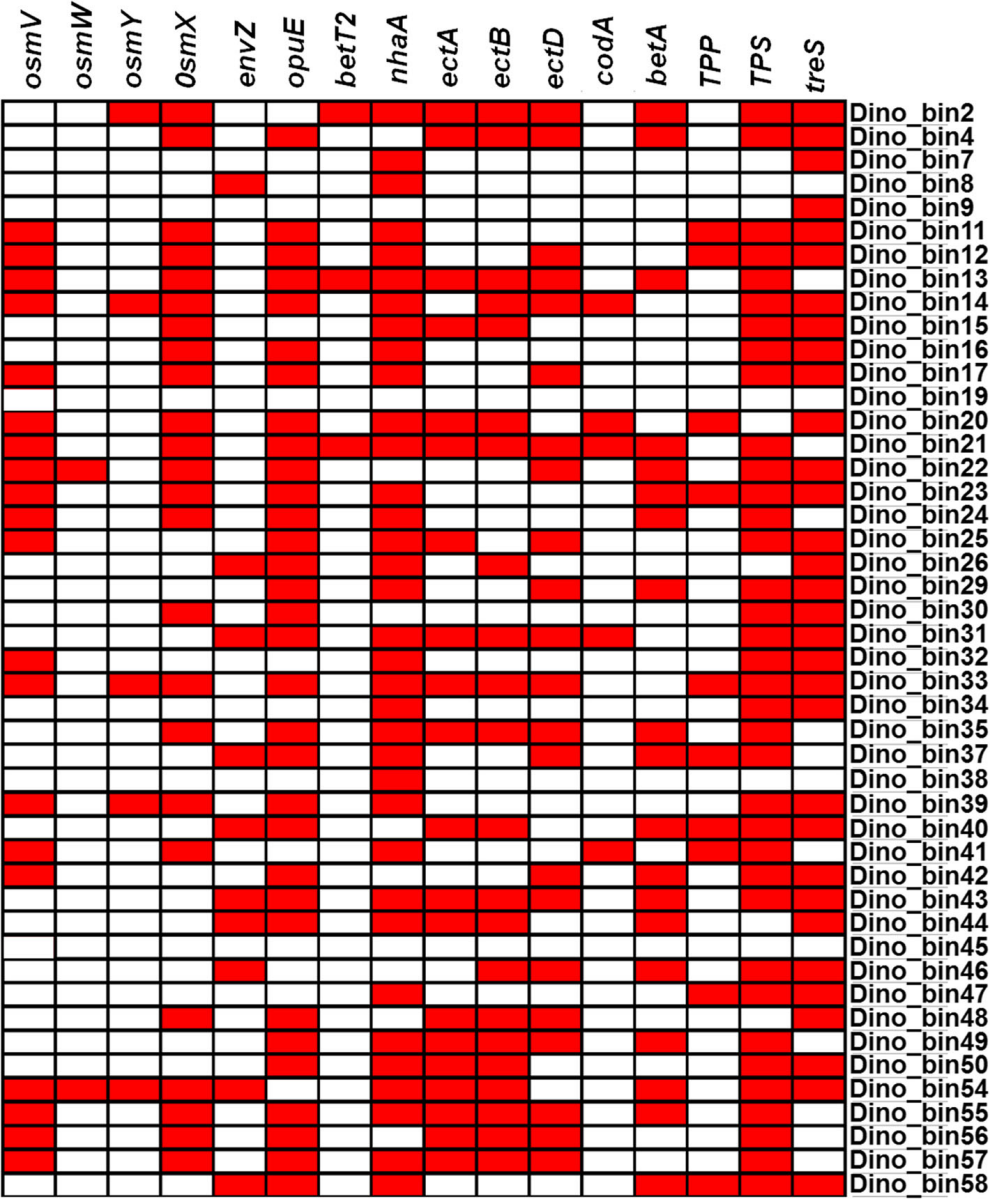
Presence of functional genes (red) in MAGs involved in coping with high osmolality in the dinosaur fossil bone. Gene abbreviations: osmolarity sensor protein (*envZ*), osmotically-inducible protein (*osmY*), osmoprotectant import ATP-binding proteins (*osmW* and *osmV*), osmoprotectant-binding protein (*osmX*), and osmoregulated proline transporter (*opuE*), osmo-dependent choline transporter (*betT2*), Na(+)/H(+) antiporter (nhaA), L-2,4-diaminobutyric acid acetyltransferase (ectA), ectoine synthase (*ectC*), ectoine hydroxylase (*ectD*), glycine-betaine producing choline oxidase (*codA*), oxygen-dependent choline dehydrogenase (*betA*), trehalose-6-phosphate phosphatase (*TPP*), trehalose-6-phosphate synthase (*TPS*) and trehalose synthase (*treS*)

Many of the MAGs harbor genetic machinery for osmoregulation, uptake and synthesis of osmoprotective compounds in order to cope with potentially high osmotic stress (Fig. 4). The genes encoding osmolarity sensor protein (*envZ*), osmotically-inducible protein (*osmY*), osmoprotectant import ATP-binding proteins (*osmW* and *osmV*), osmoprotectant-binding protein (*osmX*), and osmoregulated proline transporter (*opuE*) were identified in 42 of the 46 MAGs (Fig. 4). The compatible solutes such as glycine-betaine and ectoine are well-known osmoprotectants widely employed by diverse microorganisms [79]. Thirty-three of the 46 MAGs encode the capacity for synthesis of glycine-betaine (*codA* and *betA*) and ectoine/hydroxyectoine (*ectA*, *ectC* and *ectD*) (Fig. 4). Furthermore, two biosynthetic pathways (trehalose-6-phosphate synthase, *TPS*; and trehalose synthase, *treS*) for production of trehalose, which could also be important in adapting to high osmolality [80], were identified in 42 of the 46 MAGs (Fig. 4). The prevalence of halotolerant microorganisms and their potential capacity for coping with osmotic stress suggests either a previous exposure to saline formation water [37] or on-going high salinity SO_4_^2−^ fluctuations due to wet/dry cycles in the vadose zone.

### Carbon metabolism

**Fig. 5:**
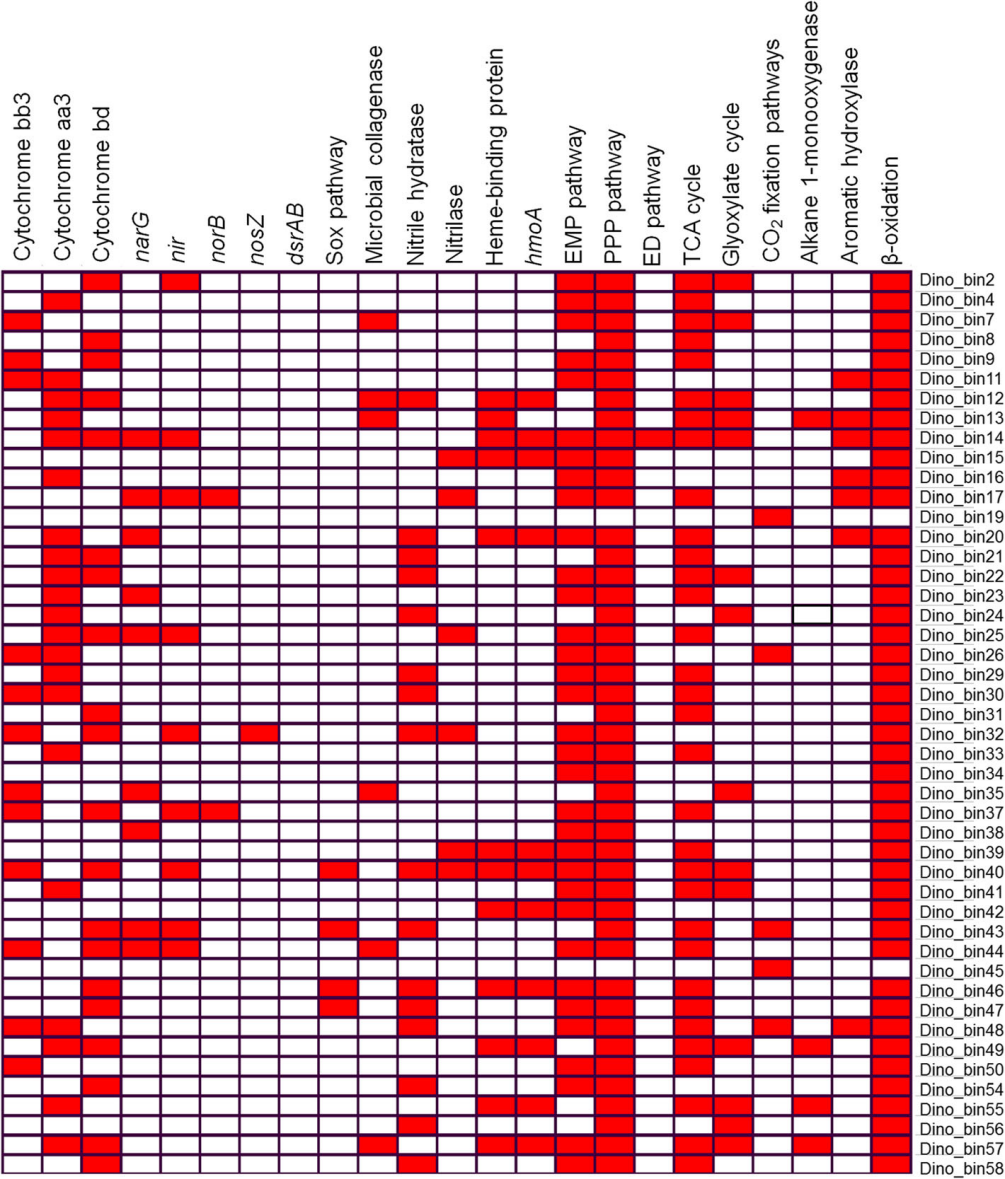
Presence of key functional genes (red) in MAGs involved in various biogeochemical cycling processes. Abbreviations: membrane bound nitrate reductase (*narG*), nitrite reductase (*nir*), nitric oxide reductase (*norB*) and nitrous oxide reductase (*nosZ*), dissimilatory sulfite reductase subunit A and B (*DsrAB*), sulfur oxidation pathway (Sox), heme-degrading monooxygenase (*hmoA*), Embden-Meyerhof pathway (EMP), pentose phosphate pathway (PPP), Entner-Doudoroff pathway (ED), tricarboxylic acid (TCA) cycle. Note: The CO_2_ fixation pathways refer to the presence of genes involved either in 3-hydroxyproprionate/4-hydroxybutyrate or reductive TCA pathway. The other pathways such as EMP and TCA indicated that majority of the associated genes (> 70%) were identified in the genome

**Fig. 6:**
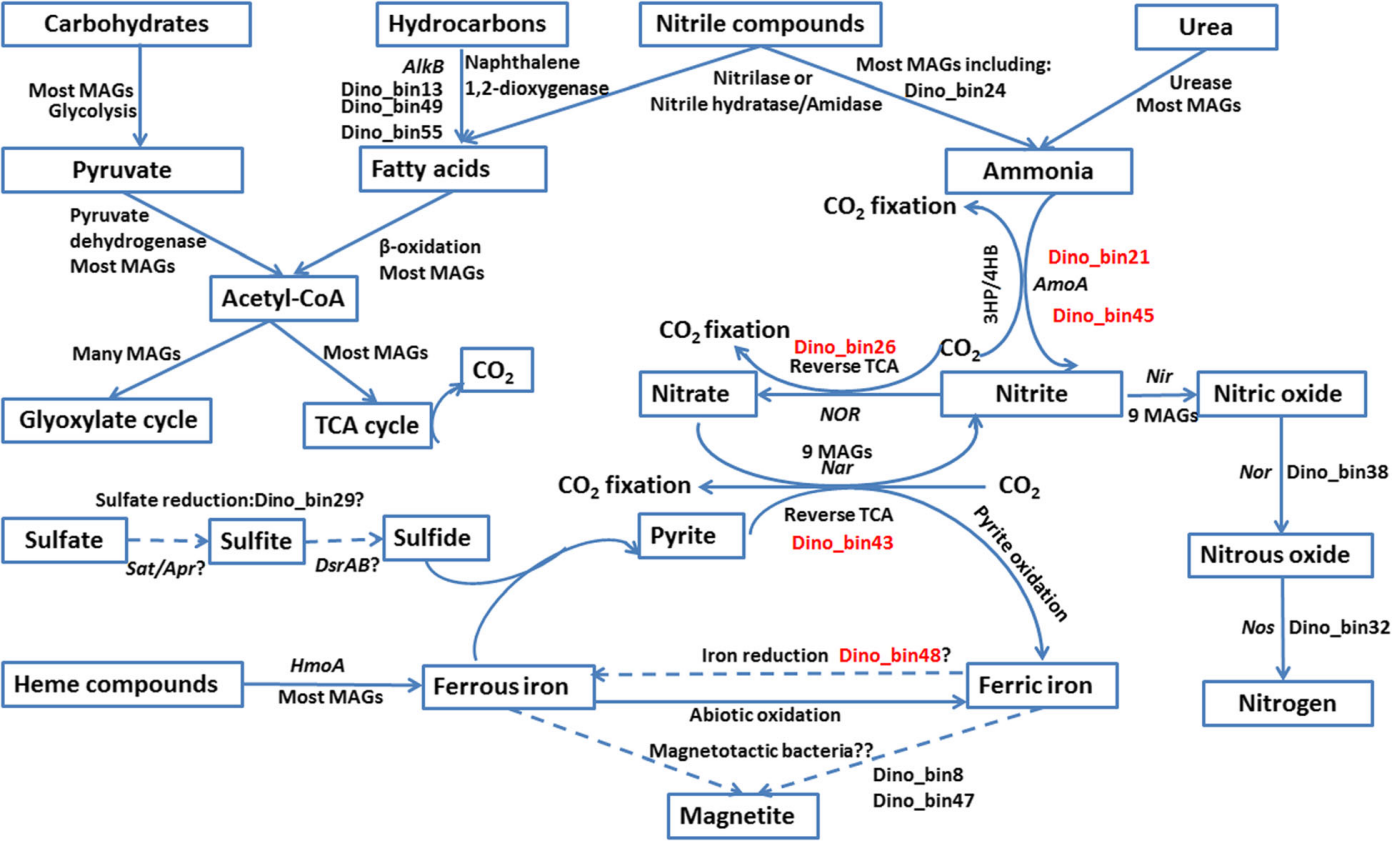
A schematic diagram showing the potential biogeochemical pathways mediated by various MAGs recovered from the *Centrosaurus* dinosaur fossil bone. The MAGs highlighted in red indicate their involvement in autotrophic CO_2_ fixation pathways and the dash lines refer to the steps where the key functional genes were not identified in the MAGs. Abbreviations: *AlkB,* alkane 1-monooxygenase; *AmoA*, ammonia monooxygenase; *narG*, membrane bound nitrate reductase; *nirS*, nitrite reductase; *Nor*, nitric oxide reductase; *nosZ*, nitrous oxide reductase; *Sat*, ATP sulfurylase; *Apr*, adenylyl-sulfate reductase; *DsrAB*, dissimilatory sulfite reductase subunit A and B; *hmoA*, heme-degrading monooxygenase; 3HP/4HB, 3-hydroxyproprionate/4-hydroxybutyrate pathway; TCA, tricarboxylic acid (TCA) cycle

Among all 46 MAGs, only 5 MAGs were predicted to harbor the key genes for CO_2_ fixation (Figs. 5 and 6). The two MAGs (Dino_bin19 and Dino_bin45) affiliated with *Thaumarchaeota* (Fig. 1) have the potential for CO_2_ fixation using the archaeal 3-hydroxyproprionate/4-hydroxybutyrate pathway that has been well described in ammonia-oxidizing *Thaumarchaeota* [81]. The *Nitrospira*-related MAG (Dino_bin_26) and two other MAGs (Dino_bin43 and Dino_bin48) encode genes for ATP-citrate lyase, 2-oxoglutarate:ferredoxin oxidoreductase and pyruvate:ferredoxin oxidoreductase that are involved in the reductive citric acid cycle for CO_2_ fixation that is found in many bacteria including *Nitrospira*-related nitrite-oxidizers [82]. The majority of the MAGs are predicted to be heterotrophs using O_2_ and/or nitrate as terminal electron acceptors (Fig. 5). Numerous genes involved in the metabolism of carbohydrates from complex polysaccharides to simple sugars were identified in most of the MAGs. For instance, many MAGs contain alpha-amylase and oligo-1,6-glucosidase that are involved in the degradation of starch and the subsequent hydrolysis of oligosaccharides. Furthermore, the genes encoding xylosidase and galactosidase were also commonly found among the MAGs. Monomeric carbon substrates such as glucose can be oxidized by the Embden-Meyerhof pathway (33 MAGs), the pentose phosphate pathway (found in 45 MAGs) and Entner-Doudoroff pathway (only Dino_bin14) (Fig. 5). The complete pathway for the tricarboxylic acid cycle was identified in 32 MAGs. By contrast, only 14 MAGs encoded the key enzymes (isocitrate lyase and malate synthase) for the glyoxylate pathway to assimilate C2 compounds like acetyl-CoA (Figs. 5 and 6) in the absence of complex substrates.

Since purported endogenous organics such as collagens and osteocytes have been discovered in various Mesozoic dinosaurs [4–10], we screened all MAGs for their potential to degrade collagens. Microbial collagenases were detected in 5 MAGs (Dino_bin7, Dino_bin12, Dino_13, Dino_bin35, and Dino_bin44) affiliated with *Actinobacteria* and *Acidobacteria* (Fig. 5), which is consistent with the ubiquity of collagen-degrading microorganisms in various soil environments [83]. Therefore, any preserved collagen peptides, if not shielded from bacterial access, could potentially be degraded by the resident microorganisms in the *Centrosaurus* bone. Previous analyses by pyrolysis-gas chromatography/mass spectrometry (Py-GC/MS) provided evidence that aromatic hydrocarbons (alkylbenzenes and naphthalenes pyrolysis products) and kerogen (n-alkane/n-alkene doublets in the pyrograms) were present in the *Centrosaurus* bone and adjacent mudstone [28]. Six MAGs (Dino_bin11, Dino_bin13, Dino_bin14, Dino_bin16, Dino_bin17, and Dino_bin20) encode genes for biphenyl 2,3-dioxygenase and naphthalene 1,2-dioxygenase to aerobically degrade aromatic hydrocarbons (Fig. 5). The alkane 1-monooxygenase gene (*alkB*) involved in the oxidation of alkanes was also found in four MAGs (Dino_bin13, Dino_bin49, Dino_bin55, and Dino_bin57) that are closely affiliated to the genus *Amycolatopsis* (Table S3 and Fig. 5), which has been implicated in the metabolism of alkanes [84]. Aside from hydrocarbon degradation, most of MAGs have the ability to further oxidize the intermediates of fatty acids via β-oxidation (Figs. 5 and 6). The β-oxidation pathway was supported by the presence of all essential genes of acyl-CoA dehydrogenase, enoyl-CoA hydratase, 3-hydroxyacyl-CoA dehydrogenase and acetyl-CoA acetyltransferase.

### Nitrogen cycling

The presence of nitrate and nitrite in the bone (Additional file 2: Table S1) suggests that nitrogen cycling might be an important biogeochemical process inside the bone microenvironments. Twenty MAGs including the dominant *Nitriliruptoria-*related MAG (Dino_bin24) encode enzymes for catalyzing hydrolysis of aliphatic and aromatic nitrile compounds (Fig. 5). Nitrile compounds are produced from biodegradation of amino acids under low O_2_ conditions [85]. A recent study has also shown that proteinaceous soft tissues can be transformed into N-heterocyclic polymers via oxidative crosslinking with lipids and polysaccharides during fossilization and diagenesis in oxic environments [86]. However, it remains unknown whether such N-rich heterocycles could be further converted to nitrile compounds during later diagenetic processes. Several MAGs associated with *Actinobacteria* were predicted to use a single-step hydrolysis, which catalyzes the hydrolysis of nitriles to yield carboxylic acids and ammonia using nitrilases (Figs. 5 and 6) [77]. Many other MAGs were predicted to hydrolyze nitriles to the same final products via a two-step hydrolysis involving nitrile hydratase and amidases (Fig. 5). Nitrile hydratase and amidases have been frequently found in diverse microorganisms from soil [87]. Notably, the iron-containing metalloenzyme nitrile hydratase from soil bacteria *Rhodococcus sp*. was also identified in a protein extract from a *Tyrannosaurus rex* bone in a previous study [11]. It remains unclear whether the expression of nitrile hydratase in dinosaur fossil bones was due to the microbial degradation of nitrile compounds that originated from the bone or the encompassing sediment/soil environment.

The ammonia produced from nitrile degradation could be used as nitrogen sources for the resident microbial community. The hydrolysis of urea catalyzed by ureases found in most of the MAGs could represent another source of ammonia. Two archaeal MAGs (Dino_bin19 and Dino_bin45) affiliated with *Thaumarchaeota* encode the metabolic capacity for ammonia oxidation by using ammonia monooxygenase (*amoA*). The nitrite could be further oxidized to nitrate by the *Nitrospira*-related MAG (Dino_bin26). Although no nitrite oxidoreductase was detected, the MAG (Dino_bin26) was phylogenetically close to other nitrite-oxidizing bacteria based on phylogenetic trees reconstructed from 16S rRNA gene (98% identity, 1538 bp) and concatenated ribosomal proteins (Figs. 1 and 3). The MAGs of 6 *Actinobacteria,* 1 *Betaproteobacteria*, 1 *Chloroflexi*, and 1 *Acidobacteria* contain *narG* (membrane bound nitrate reductase) gene for dissimilatory nitrate reduction (Fig. 5). The critical gene for nitrite reductase (*nirS)* was found in 5 *Actinobacteria*, 1 *Acidobacteria*, and 3 *Proteobacteria* MAGs (Fig. 5). The genes encoding nitric oxide reductase (*norB*) and nitrous oxide reductase (*nosZ*), were present in 3 MAGs (Dino_bin17, Dino_bin32, Dino_bin37). Although none of the MAGs contained the complete set of genes for denitrification, the presence of multiple genes suggested that the microbial populations in the bone have the genetic machinery to potentially reduce nitrate fully to N_2_ via species interaction when O_2_ becomes depleted inside the microenvironments.

### Potential sulfur and iron metabolism

Although sulfate was the most abundant electron acceptor for microbial respiration in the bone and mudstone (Additional file 2: Table S1), none of the MAGs encode the *dsrAB* gene (dissimilatory sulfite reductase) responsible for reducing sulfite to sulfide. Since O_2_ and nitrate are preferable electron acceptors over sulfate from a thermodynamic point of view, sulfide production linked to sulfate reduction appears to be less important in the *Centrosaurus* bone in this particular setting at the time of sampling. However, the common presence of framboidal pyrite in fossil bone has been interpreted as evidence that sulfate-reducing bacteria are present during fossilization in anoxic burial environments [88]. Despite the lack of *dsrAB*, the dominant *Deltaproteobacteria* MAG (Dino_bin29) in the bone was most closely related to a well-known sulfate-reducer *Desulfomonile tiedjei* [89] (Figs. 1 and 3). Therefore, the activity of this putative sulfate-reducing bacterium may have played a role in at least the recent taphonomy of the *Centrosaurus* bone. The MAGs from *Alphaproteobacteria* (Dino_bin40, Dino_bin46 and Dino_bin47) and *Betaproteobacteria* (Dino_bin43) contained the *sox* gene cluster that is involved in the sulfur oxidation pathway (Fig. 5). The *Betaproteobacteria* MAG (Dino_bin43) was particularly interesting because one of its closest relatives (*Thiobacillus denitrificans*) has been documented to couple anaerobic pyrite oxidation to nitrate reduction [90]. Therefore, the dominant *Betaproteobacteria* in the bone might cause taphonomic alternations to the fossil bone by oxidizing diagenetic pyrite in the fossil during its late diagenetic history. Furthermore, the oxidation of pyrite to sulfate coupled to nitrate reduction [90] might have contributed to the elevated sulfate concentrations in the *Centrosaurus* bone.

Iron has been previously hypothesized to play an important role in exceptional preservation of soft tissues in Mesozoic fossil bone from deep time [91], although this preservation mechanism has not been conclusively demonstrated. Iron-containing dinosaur biomolecules such as hemoglobin and myoglobin could be important original sources of iron [6], and heme-containing compounds purportedly from the breakdown of hemoglobin have been reported in trabecular bone of *Tyrannosaurus rex* [92]. Interestingly, 11 MAGs encode genes for heme-uptake proteins and heme-degrading monooxygenase. Therefore, any endogenous heme-containing compounds from the *Centrosaurus* could have been potentially degraded by the resident microbes to liberate iron into the microenvironments. Many types of iron minerals including iron oxides, iron-bearing carbonates and pyrite form in fossil bones during diagenesis depending upon pH and redox conditions [17, 93]. As discussed earlier, pyrite can be oxidized to ferric iron by the *Thiobacillus denitrificans*-like microorganism (Dino_bin43) when coupled to nitrate reduction [90]. On the other hand, Fe(III) oxides can be used as the terminal electron acceptors by iron-reducing bacteria and multiheme cytochromes have been implicated in ferric iron reduction [94]. Although the *Acidimicrobiia*-related MAG (Dino_bin48) was closely related to an iron-reducer *Aciditerrimonas ferrireducens* [95] (Fig. 1), cytochromes containing multiple heme-binding motifs were not found in the genome**.** Therefore, more information would be needed to show whether Fe(III) oxides in the dinosaur bone could possibly be reduced when anoxic conditions prevail in the microenvironments. More intriguingly, two *Alphaproteobacteria* MAGs (Dino_bin8 and Dino_bin47) were phylogenetically close to magnetotactic bacteria such as *Magnetospirillum marisnigri* [96] and *Magnetospirillum gryphiswaldense* [97] (Fig. 1). These magnetotactic bacteria can accumulate iron to form magnetosomes used to sense the ambient geomagnetic field direction.. However, the genes encoding magnetosome-related proteins were not identified in these two MAGs (Dino_bin8 and Dino_bin47). Therefore, the role of magnetotactic bacteria in iron uptake and magnetite biomineralization in dinosaur fossil bones warrants further investigation.

### Metaproteomics and in situ replication rates

Mass spectrometry has been employed to identify collagen peptides from Mesozoic dinosaur fossils [5, 8], although their origin is controversial [13, 14]. Metaproteomes were generated from proteins extracted from the *Centrosaurus* bone and adjacent mudstone using four different methods (see Methods). To search for the existence of endogenous collagen peptides, the obtained metaproteomic datasets were searched against a customized database composed of the Uniprot Vertebrates database and collagen peptides reported from *Tyrannosaurus rex* [11] and *Brachylophosaurus canadensis* [8]*.* No collagen-related peptides were identified in either the *Centrosaurus* bone or the adjacent mudstone. The failure to detect endogenous collagen peptides from the fossil bone was consistent with our previous results using other analytical methods such as amino acid racemization analysis and Py-GC/MS [28]. Further metaproteomic analyses using a database constructed from the proteomes of all the MAGs indicate that the identified peptide sequences represented proteins expressed from most of the microorganisms inhabiting the bone and mudstone. Due to the much higher microbial biomass inside the bone relative to that of the mudstone [28], more proteins were extracted and identified from the bone (401) than the mudstone (187) (Additional file 7: Table S6 and Additional file 8: Table S7). Although the number of identified proteins varied among all MAGs (Additional file 7: Table S6 and Additional file 8: Table S7), the number of identified proteins was highest among the MAGs (Dino_bin24, Dino_bin20, Dino_bin22 and Dino_bin31 in Additional file 1: Fig. S9) that are abundant in the fossil bone (Fig. 2). Therefore, if any endogenous dinosaur peptides were present, such overwhelming signals from bacteria could complicate their identification and interpretation.

Due to the poor efficiency of protein recovery from the *Centrosaurus* bone, the number of identified proteins from each MAG was very limited (Additional file 1: Fig. S9). Enzymes involved in β-oxidation such as acyl-CoA dehydrogenase and enoyl-CoA hydratase were synthesized from several MAGs including the dominant *Deltaproteobacteria* MAG (Dino_bin29) in the *Centrosaurus* bone (Fig. 3). NADH-quinone oxidoreductases involved in the aerobic respiratory chains were expressed from several MAGs and thus confirmed the aerobic heterotrophic metabolism among the microbial populations in the *Centrosaurus* bone. Aromatic hydroxylases involved in the metabolism of naphthalene (1-hydroxy-2-naphthoate) and benzoate (benzoate-CoA ligase) were also identified in some of the MAGs. The metaproteomic data also confirmed that nitrile hydratase was synthesized in situ in one of the *Alphaproteobacteria* MAGs (Dino_bin58). As mentioned earlier, nitrile hydratase associated with *Actinobacteria* (*Rhodococcus sp.*) was identified from the fossil bone of *Tyrannosaurus rex* [11]. In addition, the gene (*betB*) involved in biosynthesis of the osmoprotectant glycine betaine was expressed from an *Actinobacteria* MAG (Dino_bin14) (Additional file 7: Table S6). Furthermore, the in situ synthesis of cold shock proteins were identified from many MAGs, suggesting the bone-bearing horizon might have been subject to rapid-onset low temperature due to the wide diurnal temperature range in this area or fast-moving cold fronts. Most of the other identified proteins were either key enzymes involved in biosynthetic pathways (fatty acids and amino acids) or hypothetical proteins with unknown function (Additional file 7: Table S6 and Additional file 7: Table S7).

Since the metaproteomics revealed microbial activity in the *Centrosaurus* bone and adjacent mudstone, the in situ replication rates of the microbial populations were estimated from metagenomic data using a recently proposed Growth Rate Index (GRiD) [62]. The GRiD values among MAGs were estimated to be in the range of 1.03 and 1.37 and a high correlation (r^2^ = 0.81) was found between the GRiD values of MAGs within the *Centrosaurus* bone and those of the same MAGs in the adjacent mudstone (Additional file 1: Fig. S10). These GRiD values are similar to the 1 to 1.5 values reported for bacteria from a shallow groundwater site in Colorado [62]. Therefore, the low GRiD values of MAGs within the *Centrosaurus* bone and mudstone seem consistent with a shallow subsurface environment likely due to limited nutrients fluxes in the *Centrosaurus* bone and surrounding mudstone. Furthermore, no good correlation was observed between GRiD values and relative abundance of MAGs (Fig. 2 and Additional file 1: Fig. S10). For example, the GRiD values from the dominant MAGs affiliated with *Euzebya* (Dino_bin24), *Deltaproteobacteria* (Dino_bin29), *Betaproteobacteria* (Dino_bin43) in the *Centrosaurus* bone were much lower than the less abundant populations from *Actinobacteria* MAGs (Dino_bin17 and Dino_bin48) (Additional file 5: Table S4 and Fig. S10). Since the GRiD values only provide a snapshot of the in situ growth rate at time of collection*,* the greater biomass abundance of MAGs in the bone relative to those in the mudstone might be attributed to faster growth rates incurred during favorable conditions in the past. Nevertheless, the metaproteomics and the estimated replication rates further support that the dominantly heterotrophic microbes that have colonized the *Centrosaurus* bone at some point prior to excavation are metabolically active in the subterranean environment [28].

### Implications for the preservation of endogenous organics in Mesozoic dinosaurs

The *Centrosaurus* bone microbial community does contain the functional potential to degrade collagen, heme-compounds, N-heterocyclic polymers, and kerogen (Fig. 6), which have been previously reported in fossil bones [4, 40, 86, 92]. All peptide sequences recovered from the *Centrosaurus* bone were microbial and none matched purported dinosaur or other vertebrae collagen. This contrasts with the metaproteome reported for a *T. rex* that recovered only a few microbial peptides [11]. Despite the high microbial biomass in the *Centrosaurus* bone, ~ 5 × 10^8^ cells/g [28], the protein recovery utilizing four different extraction techniques was low. If the characteristics we reported here (i.e. high microbial abundance combined with the enzymatic repertoire to degrade endogenous compounds) are symptomatic of fossil bones near the surface, then it appears that the only way collagens in dinosaur bone could survive is if they were protected from microbial degradation during their entire taphonomic history. A negative fluorescent staining result from an immunoassay targeting peptidoglycan, a common bacterial cell wall constituent, might indicate the microbial abundance in the soft tissue fraction of a *B. canadensis* and *T. rex* bone was much less than that of ancient organics of endogenous origins [98]. Even if portions of dinosaur bones remained sealed to micribial colonization, a mechanism is still required to substantively reduce the in situ rate of protein hydrolysis. Because nucleic acids and proteins can be extracted from soft tissue simultaneously and because sequencing is rather affordable, the combined approaches presented here could offer important clues to the remaining questions surrounding the exceptional preservation of dinosaur soft tissues in future studies.

## Conclusion

Combined geochemical, metagenomic and metaproteomic analyses of an excavated *Centrosaurus* bone and encompassing mudstone revealed a cm-scale bone microenvironment with greater microbial abundance than that of the mudstone, which is likely due to the higher phosphate content of the bone. The *Centrosaurus* bone microbial community is distinct from that of the encompassing mudstone, which may relate to the higher concentrations of nitrate and sulfate. By using genome-resolved metagenomics, we reconstructed 46 draft genomes (MAGs) that included bacterial members of *Nitriliruptoria*, *Deltaproteobacteria*, *Betaproteobacteria* and *Acidimicrobiia* who were more abundant in the *Centrosaurus* bone than in the adjacent mudstone. The majority of the recovered MAGs represented novel taxonomic lineages, previously characterized only by 16S rRNA gene sequences. Metagenomic and metaproteomic analyses indicate that the bone microbiome is primarily comprised of active, though slowly growing, aerobic heterotrophs capable of oxidizing a wide range of organic substrates and nitrogenous compounds consistent with the vadose zone environment of the bone. The *Centrosaurus* bone does contain a mixture of organic compounds [28], some of which are radiocarbon-dead hydrocarbons that could have migrated from underlying gas reservoirs [35] (although a contribution from endogenous, organic geopolymers is possible) and some of which are radiocarbon-active, potentially humic and fulvic acids from recharging meteoric water [99]. The metagenomic data also indicated that the bone and mudstone communities are capable of nitrate reduction under sub-oxic conditions that might prevail under water saturation. Collectively, our genomic-centric analyses revealed that the novel lineages residing in the bone might be involved in the interconnected biogeochemical processes linked to metabolism of carbon, sulfur, nitrogen and iron (Fig. 6). Therefore, the metabolic activity of bone microbiome can cause taphonomic alterations to the fossil bone throughout geological time. In this regard, the prevalence of microbial life in Mesozoic fossils should be considered when searching for endogenous fossil organics preserved through deep time. The fact that these microbes can metabolize diagenetically unstable organics such as collagen protein, as well as thermally stable organics such as kerogen and humic-like nitrogenous polymers, is of concern in the attempt to assume high proportions of protein- or labile lipid-derived organics in open systems such as fossil bones are endogenous. Examinations should be done on dinosaur bones with different depositional setting, stratigraphy, palaeoclimate and paleogeography to determine whether a relationship exists between fossil bone preservation and the microbial inhabitants, and if living microbial community composition and metabolism are better explained by modern climate and environmental conditions.

## Supplementary information

**Additional file 1: Figure S1.** Canonical correspondence analysis (CCA) for microbial community and geochemical variables in the *Centrosaurus* bone (1B5g1 and 1B5g2), EDTA-treated bone (1BEDTA and 1BEDTA2), bone scrapings (1S1 and 1S2) and adjacent mudstone (1M1 and 1M2). Arrows indicate the direction and magnitude of environmental parameters associated with samples (dots) and major bacterial groups on class level (triangles). **Figure S2.** Selected dominant groups within microbial community from bone and mudstone as determined by 16S rRNA amplicon sequencing (A) and genome-resolved metagenomics (B). The relative abundance in A and B was based on the number of OTUs and coverage of MAGs, respectively. The asterisks indicated the statistical difference (*<0.05 and **<0.01) between the bone (1B5g) and mudstone and mudstone (1M10g). **Figure S3.** Pairwise average amino acid identity (AAI) distances among the *Euzebya*-related MAGs (Dino_bin24 and Dino_bin30) and their closest genomic relatives. **Figure S4.** Pairwise average amino acid identity (AAI) distances among the *Betaproteobacteria*-related MAG (Dino_bin43) and its closest genomic relatives. **Figure S5.** Pairwise average amino acid identity (AAI) distances among the *Deltaproteobacteria*-related MAG (Dino_bin29) and its closest genomic relatives. **Figure S6.** Pairwise average amino acid identity (AAI) distances among the *Chloroflexi*-related MAGs and their closest genomic relatives. **Figure S7.** Pairwise average amino acid identity (AAI) distances among the *Acidimicrobiia*-related MAGs and their closest genomic relatives. **Figure S8.** Pangenomics analysis of the *Euzebya-related* MAGs and other publically available genomes of rare *Actinobacteria* associated with the class *Nitriliruptoria.* Three gene clusters that are only present in *Euzebya-related* MAG from the dinosaur bone are highlighted as 1, 2, and 3 in red. The two MAGs (Dino_bin24 and Dino_bin30) in this study were highlighted in blue whereas other genomes related to *Nitriliruptoria* were shown in purple. The core genes refer to the genes that are shared by all species. **Figure S9.** Number of expressed proteins identified from each MAG in the dinosaur fossil bone (1B5g) and the adjacent mudstone (1M10g). The number was based on the total proteins identified from the 8 metaproteomeic datasets generated from proteins extracted with different approaches. **Figure S10.** GRiD measurement of bacterial MAGs from the metagenomic datasets from ***Centrosaurus*** bone and adjacent mudstone. The data points indicate the GRiD values of MAGs that the **dnaA/ori** and **ter/dif** ratios were above 0.8 according to the output results from the GRiD tool.

**Additional file 2: Table 1.** Geochemical characteristics in water extract from the bone and mudstone.

**Additional file 3: Table S2.** Statistical summary for MAGs recovered from microbiome in dinosaur fossil bone.

**Additional file 4: Table S3.** Taxonomic classification based on the GTDB-Tk tool.

**Additional file 5: Table S4.** Relative abundance of MAGs in each metagenome

**Additional file 6: Table S5** Taxonomic identification of MAGs based on homology of 16S rRNA gene.

**Additional file 7: Table S6.** Identified proteins in dinosaur fossil bone

**Additional file 8: Table S7.** Identified proteins in the adjacent mudstone.

**Additional file 9. **Accession numbers for individual MAGs deposited in NCBI database.

## Data Availability

All metagenomic reads and sample information are available at NCBI under BioProject ID PRJNA494230 with the SRA accession numbers of SRR10173838 and SRR10173105. Accession numbers for individual MAGs are provided in Additional file 9: Table S8. All metaproteomic data and identification results were deposited to the ProteomeXchange Consortium via the PRIDE partner repository with the dataset identifier PXD015750.
