# Supplementary material for: Genome-centric resolution of novel microbial lineages in an excavated Centrosaurus dinosaur fossil bone from the Late Cretaceous of North America
Source: Environ Microbiome. 2020 Mar 19;15:8. doi: 10.1186/s40793-020-00355-w (PMC8067395; doi:10.1186/s40793-020-00355-w)
Supplement: Supplementary file 1 — Additional file 1: Figure S1. Canonical correspondence analysis (CCA) for microbial community and geochemical variables in the Centrosaurus bone (1B5g1 and 1B5g2), EDTA-treated bone (1BEDTA and 1BEDTA2), bone scrapings (1S1 and 1S2) and adjacent mudstone (1M1 and 1M2). Arrows indicate the direction and magnitude of environmental parameters associated with samples (dots) and major bacterial groups on class level (triangles). Figure S2. Selected dominant groups within microbial community from bone and mudstone as determined by 16S rRNA amplicon sequencing (A) and genome-resolved metagenomics (B). The relative abundance in A and B was based on the number of OTUs and coverage of MAGs, respectively. The asterisks indicated the statistical difference (*<0.05 and **<0.01) between the bone (1B5g) and mudstone and mudstone (1M10g). Figure S3. Pairwise average amino acid identity (AAI) distances among the Euzebya-related MAGs (Dino_bin24 and Dino_bin30) and their closest genomic relatives. Figure S4. Pairwise average amino acid identity (AAI) distances among the Betaproteobacteria-related MAG (Dino_bin43) and its closest genomic relatives. Figure S5. Pairwise average amino acid identity (AAI) distances among the Deltaproteobacteria-related MAG (Dino_bin29) and its closest genomic relatives. Figure S6. Pairwise average amino acid identity (AAI) distances among the Chloroflexi-related MAGs and their closest genomic relatives. Figure S7. Pairwise average amino acid identity (AAI) distances among the Acidimicrobiia-related MAGs and their closest genomic relatives. Figure S8. Pangenomics analysis of the Euzebya-related MAGs and other publically available genomes of rare Actinobacteria associated with the class Nitriliruptoria. Three gene clusters that are only present in Euzebya-related MAG from the dinosaur bone are highlighted as 1, 2, and 3 in red. The two MAGs (Dino_bin24 and Dino_bin30) in this study were highlighted in blue whereas other genomes related to Nitriliruptoria we [file 40793_2020_355_MOESM1_ESM.docx]

**Supporting Information for:**

**Genome-centric resolution of novel microbial lineages in an excavated *Centrosaurus* dinosaur fossil bone from the Late Cretaceous of North America**

Renxing Liang^1*^, Maggie C.Y. Lau^1#^, Evan T. Saitta^2^, Zachary K. Garvin^1^, Tullis C. Onstott^1^

^1^Department of Geosciences, Princeton University, Princeton, United States

^2^Integrative Research Center, Section of Earth Sciences, Field Museum of Natural History, Chicago, United States

**^*^For correspondence**

**E-mail:** [rliang@princeton.edu](mailto:rliang@princeton.edu)

**Mailing address:** B88, Guyot Hall, Princeton University, Princeton, NJ, USA, 08544

**Tel:** +1-609-258-2387

^#^Present address: Institute of Deep-Sea Science and Engineering, Chinese Academy of Sciences, Sanya, China

**This additional information contains:**

- - 10 Pages
  - 10 Figures


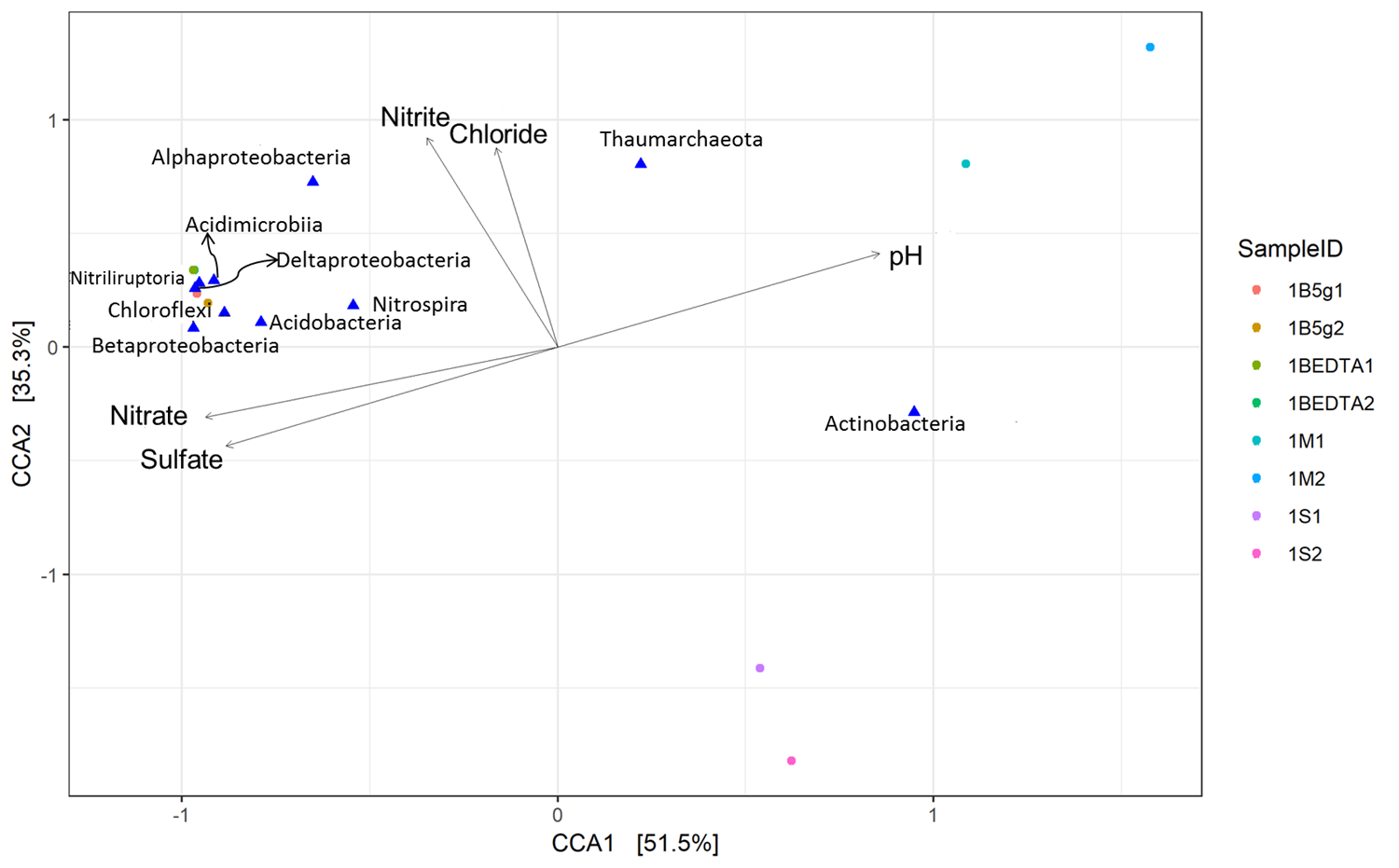


**Figure S1.** Canonical correspondence analysis (CCA) for microbial community and geochemical variables in the *Centrosaurus* bone (1B5g1 and 1B5g2), EDTA-treated bone (1BEDTA and 1BEDTA2), bone scrapings (1S1 and 1S2) and adjacent mudstone (1M1 and 1M2). Arrows indicate the direction and magnitude of environmental parameters associated with samples (dots) and major bacterial groups on class level (triangles).


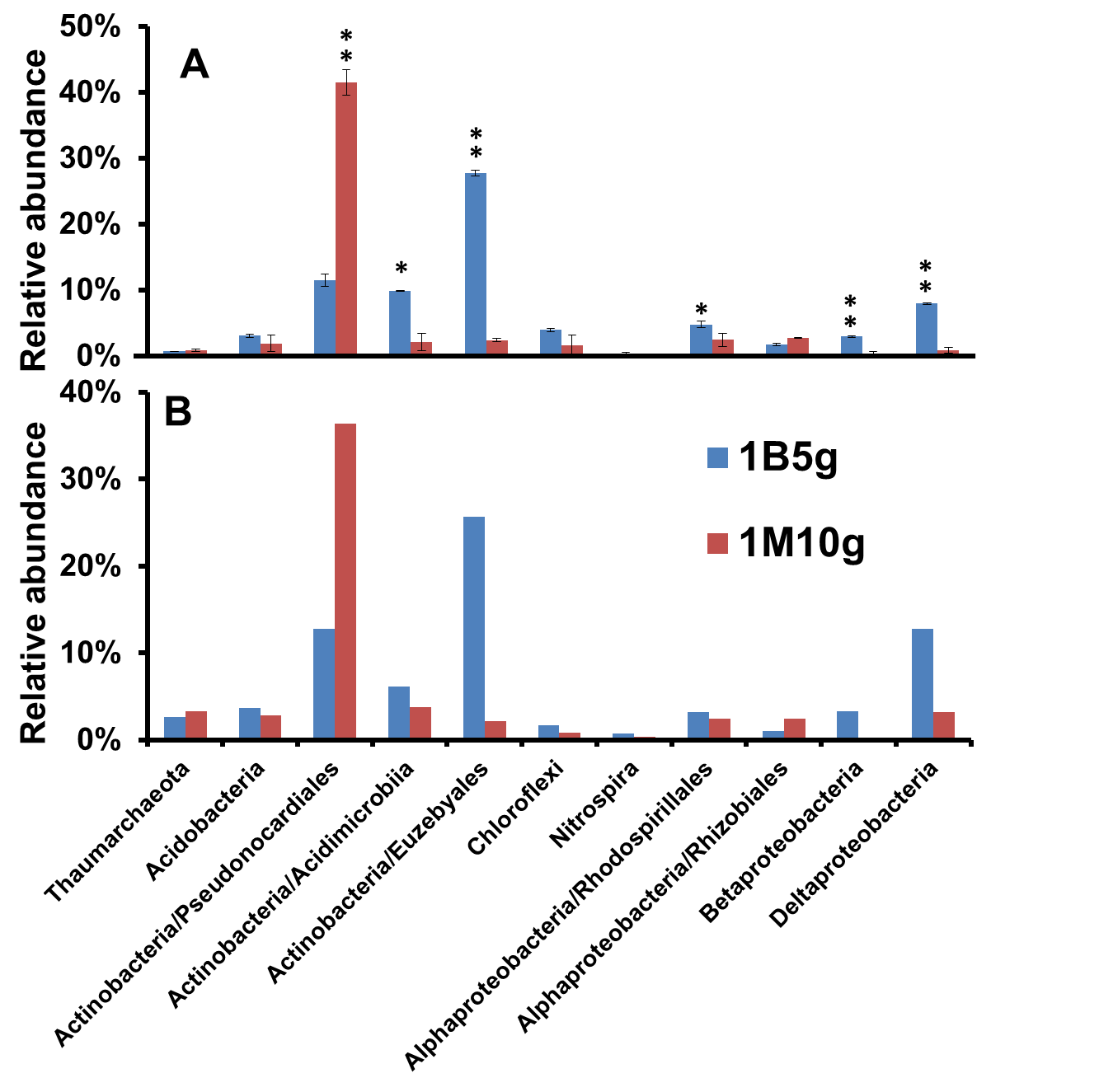


**Figure S2.** Selected dominant groups within microbial community from bone and mudstone as determined by 16S rRNA amplicon sequencing (A) and genome-resolved metagenomics (B). The relative abundance in A and B was based on the number of OTUs and coverage of MAGs, respectively. The asterisks indicated the statistical difference (* <0.05 and **<0.01) between the bone (1B5g) and mudstone and mudstone (1M10g).


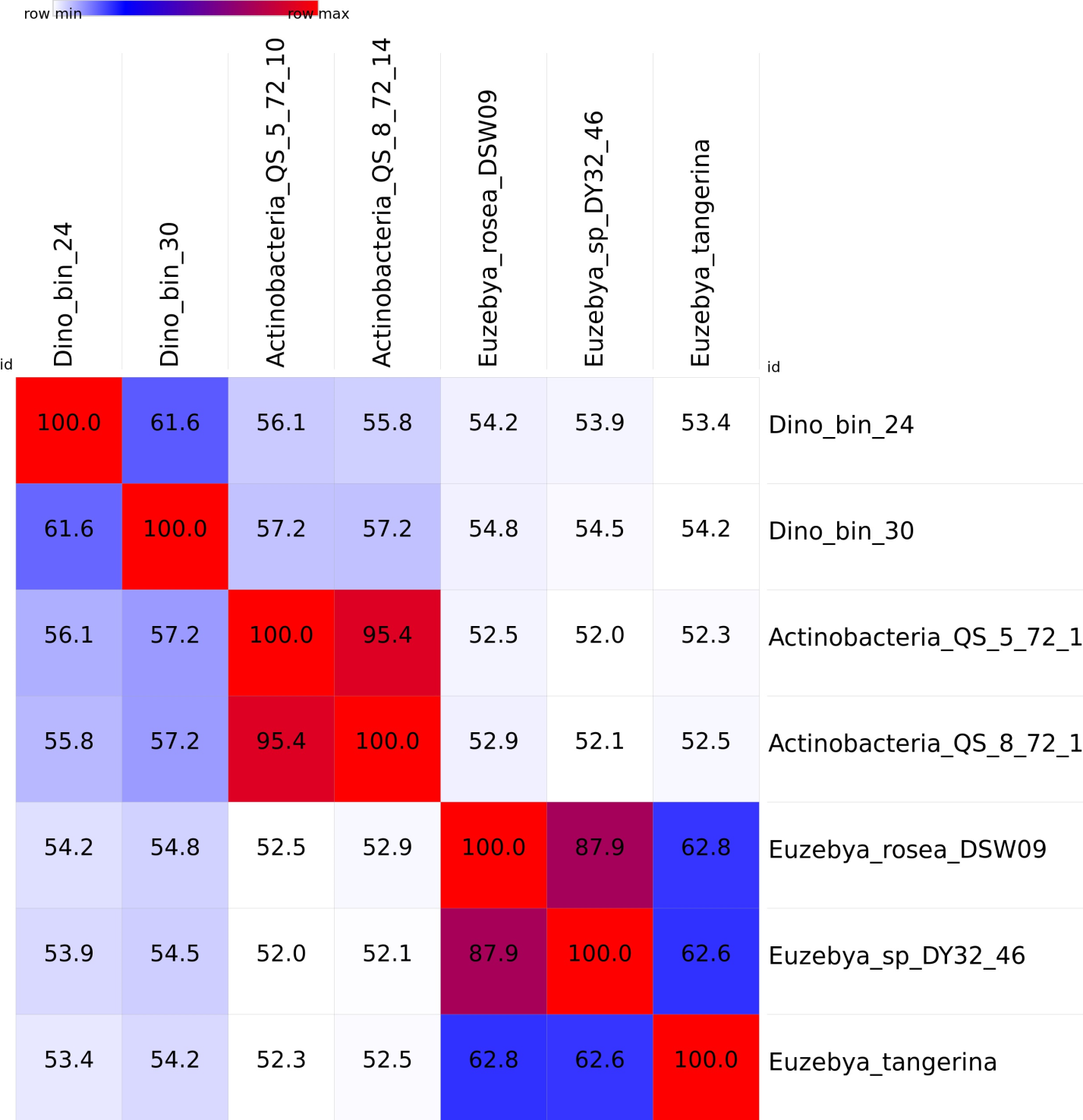


**Figure S3** Pairwise average amino acid identity (AAI) distances among the *Euzebya*-related MAGs (Dino_bin24 and Dino_bin30) and their closest genomic relatives.


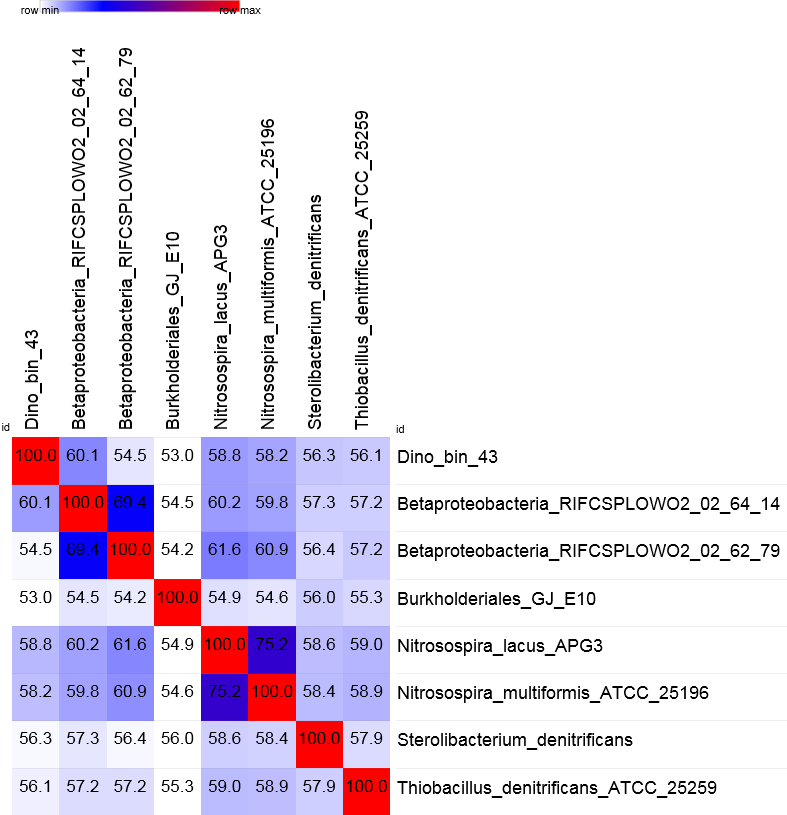


**Figure S4** Pairwise average amino acid identity (AAI) distances among the *Betaproteobacteria*-related MAG (Dino_bin43) and its closest genomic relatives.


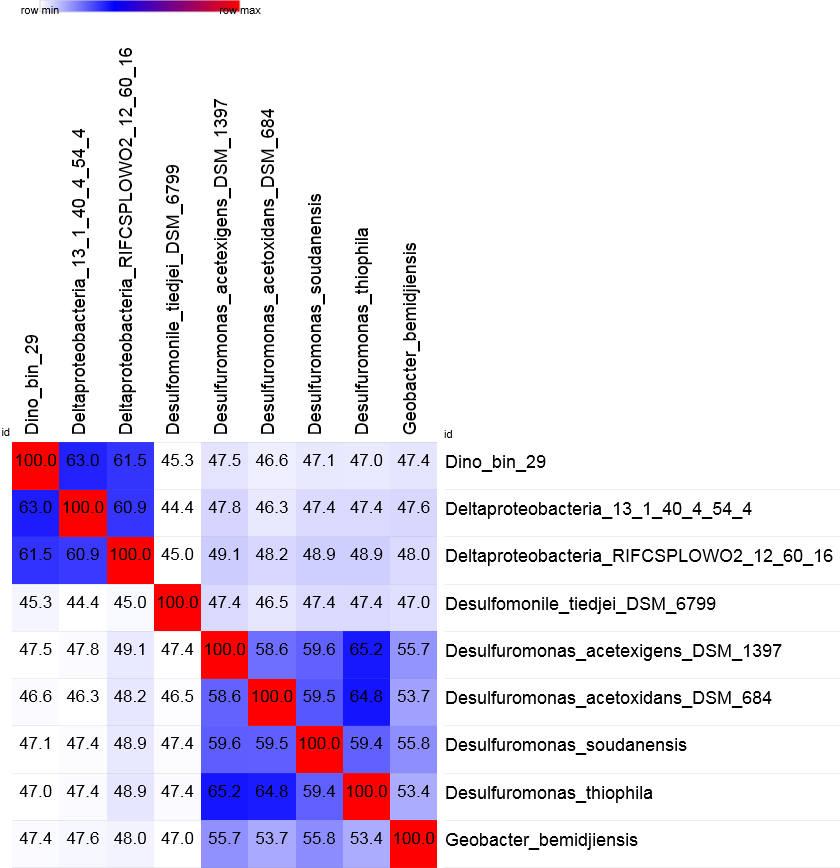


**Figure S5** Pairwise average amino acid identity (AAI) distances among the *Deltaproteobacteria*-related MAG (Dino_bin29) and its closest genomic relatives.


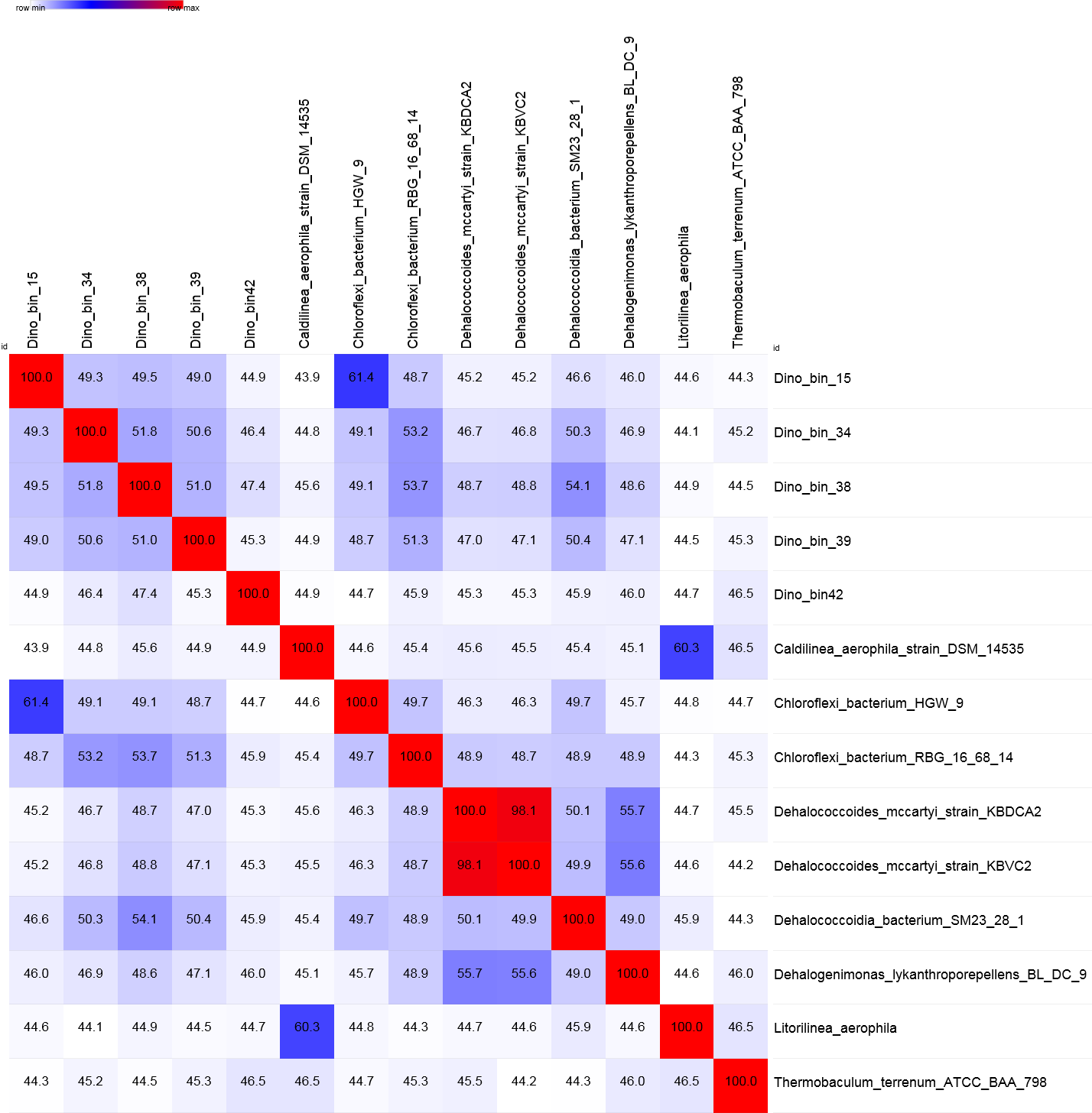


**Figure S6** Pairwise average amino acid identity (AAI) distances among the *Chloroflexi*-related MAGs and their closest genomic relatives.


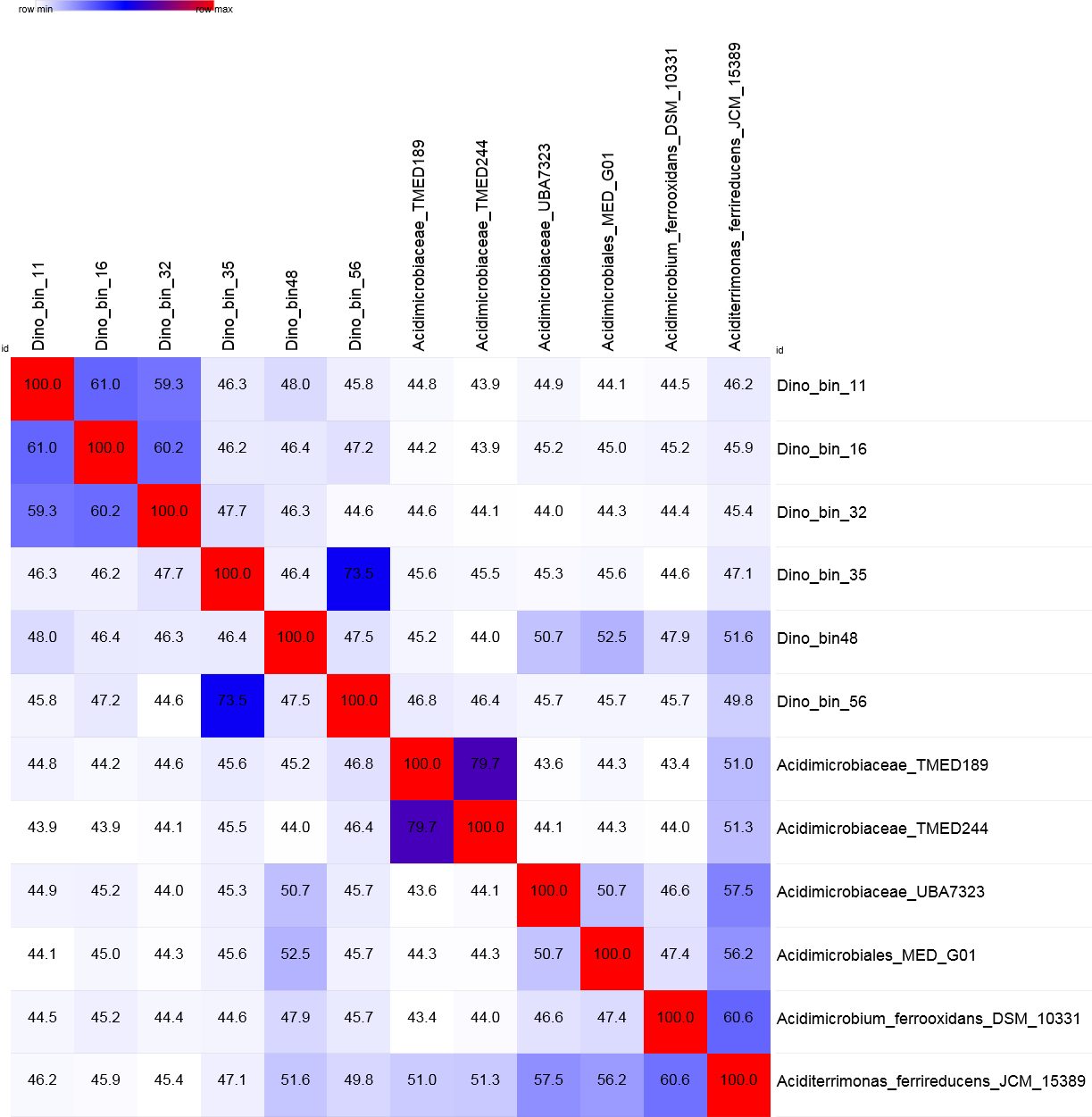


**Figure S7** Pairwise average amino acid identity (AAI) distances among the *Acidimicrobiia*-related MAGs and their closest genomic relatives.

**
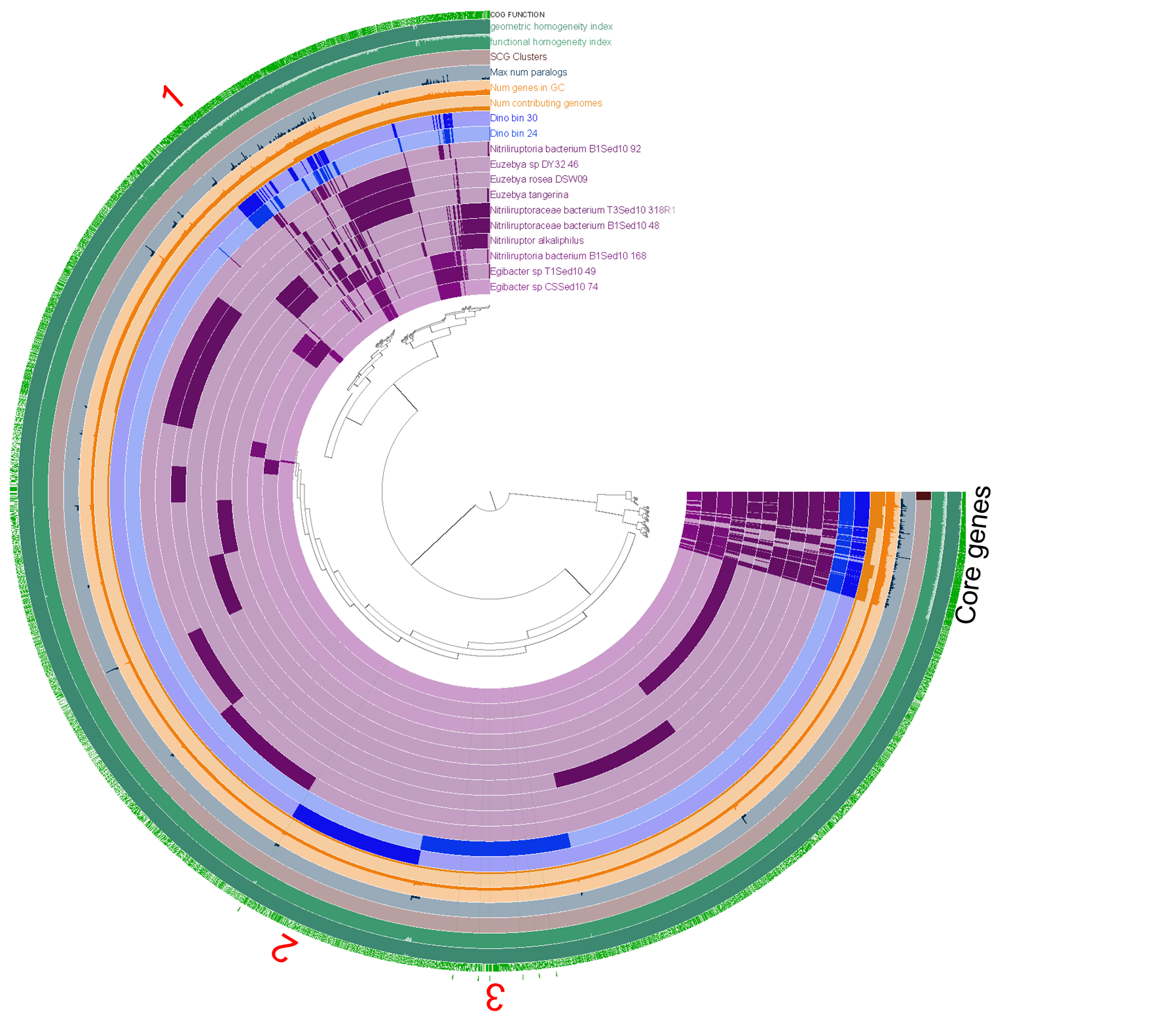
**

**Figure S8** Pangenomics analysis of the *Euzebya-related* MAGs and other publically available genomes of rare *Actinobacteria* associated with the class *Nitriliruptoria.* Three gene clusters that are only present in *Euzebya-related* MAG from the dinosaur bone are highlighted as 1, 2, and 3 in red. The two MAGs (Dino_bin24 and Dino_bin30) in this study were highlighted in blue whereas other genomes related to *Nitriliruptoria* were shown in purple. The core genes refer to the genes that are shared by all species.


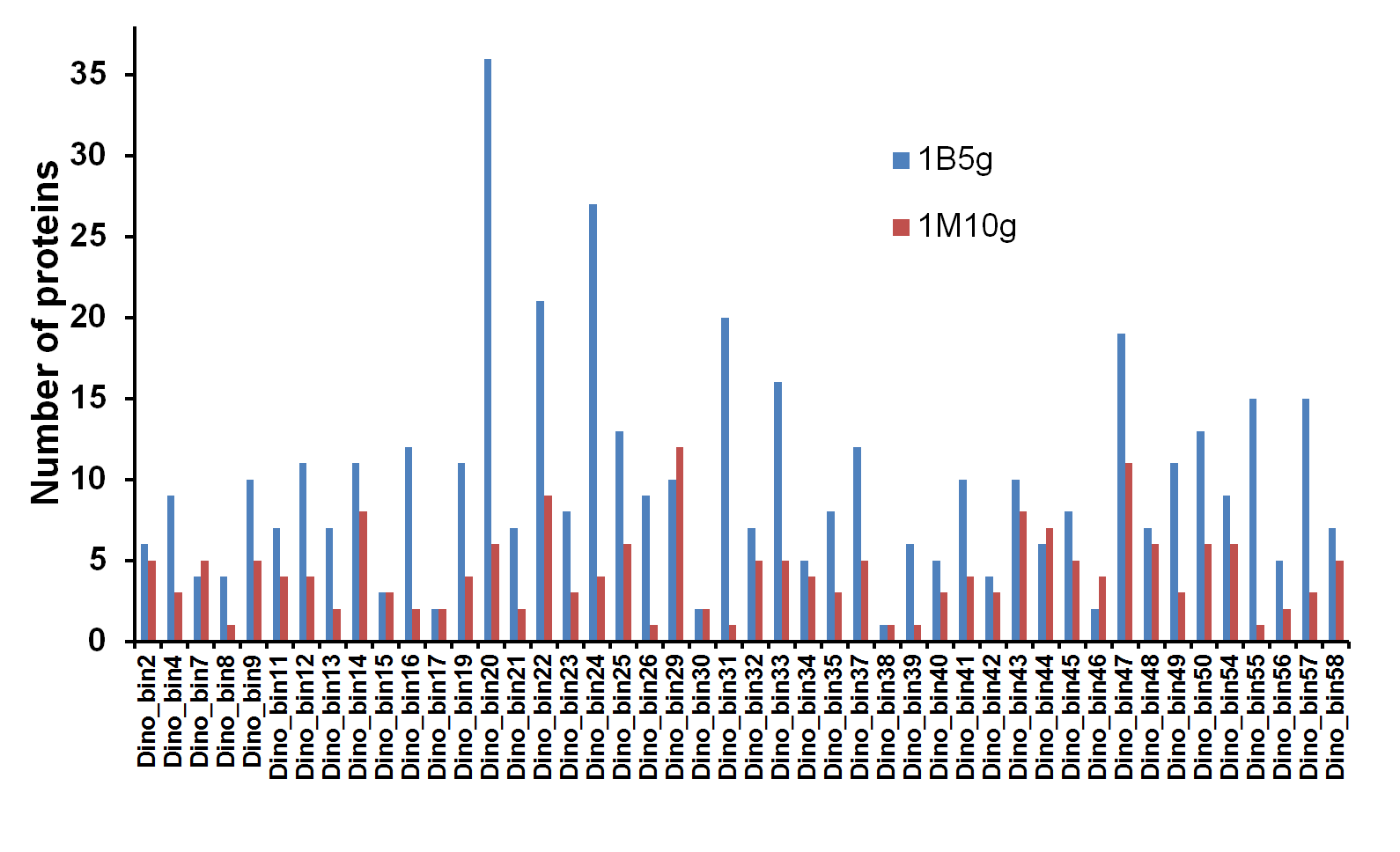


**Figure S9** Number of expressed proteins identified from each MAG in the dinosaur fossil bone (1B5g) and the adjacent mudstone (1M10g). The number was based on the total proteins identified from the 8 metaproteomeic datasets generated from proteins extracted with different approaches.


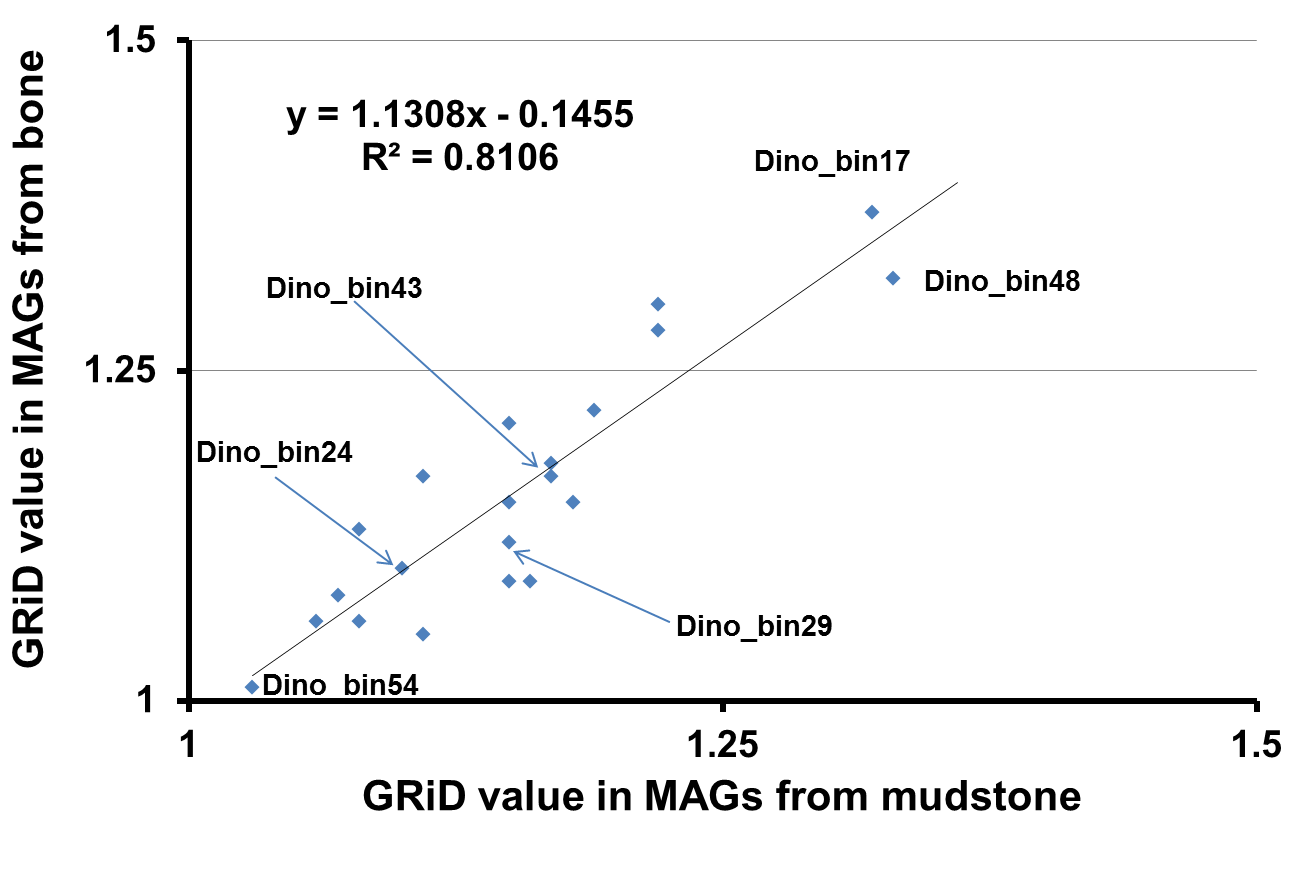


**Figure S10** GRiD measurement of bacterial MAGs from the metagenomic datasets from ***Centrosaurus*** bone and adjacent mudstone. The data points indicate the GRiD values of MAGs that the dnaA/ori and ter/dif ratios were above 0.8 according to the output results from the GRiD tool.
