# Supplementary material for: Genome-centric resolution of novel microbial lineages in an excavated Centrosaurus dinosaur fossil bone from the Late Cretaceous of North America
Source: Environ Microbiome. 2020 Mar 19;15:8. doi: 10.1186/s40793-020-00355-w (PMC8067395; doi:10.1186/s40793-020-00355-w)
Supplement: Supplementary file 2 — Additional file 2: Table 1. Geochemical characteristics in water extract from the bone and mudstone. [file 40793_2020_355_MOESM2_ESM.docx]

| Sample type | pH | F^-^  ^(µg/g)^ | Cl^-^  ^(µg/g)^ | SO_4_^2-^  ^(µg/g)^ | PO_4_^3-^  ^(µg/g)^ | NO_2_^-^  ^(µg/g)^ | NO_3_^-^  ^(µg/g)^ | Organic acids^2^  ^(µg/g)^ |
| --- | --- | --- | --- | --- | --- | --- | --- | --- |
| Bone | 5.5 | 7.9±0.5 | 6.1±0.5 | 4910±24 | 5.8±1.8 | 12.1±0.6 | 1210±7 | BDL |
| Mudstone | 6.5 | 5.6±0.3 | 6.2±1.1 | 546±2 | BDL^1^ | 11.9±0.1 | 575.2±0.4 | BDL |
| Scrapings | 6 | 4.9±0.4 | 4.2±0.3 | 4000±180 | BDL | 6.1±0.1 | 986±38 | BDL |

**Table 1** Geochemical characteristics in water extract from the bone and mudstone.

^1^ BDL, below detection limit. The lowest detection limit for PO_4_^3-^ was 3 µg/g.

^2^ The lowest detection limit for organic acids (formate, acetate, and propionate) was 1.3 µg/g.
