# Supplementary material for: Genome-centric resolution of novel microbial lineages in an excavated Centrosaurus dinosaur fossil bone from the Late Cretaceous of North America
Source: Environ Microbiome. 2020 Mar 19;15:8. doi: 10.1186/s40793-020-00355-w (PMC8067395; doi:10.1186/s40793-020-00355-w)
Supplement: Supplementary file 3 — Additional file 3: Table S2. Statistical summary for MAGs recovered from microbiome in dinosaur fossil bone. [file 40793_2020_355_MOESM3_ESM.docx]

| MAGs | Completeness (%) | Contamination (%) | GC (%) | Number of CDs | N50  (kb) | Size (Mb) | Number of scaffolds |
| --- | --- | --- | --- | --- | --- | --- | --- |
| Dino_bin2 | 99.29 | 2.14 | 72.27 | 5966 | 23.68 | 6.09 | 413 |
| Dino_bin4 | 94.82 | 1.84 | 70.63 | 4461 | 10.39 | 4.65 | 632 |
| Dino_bin7 | 91.29 | 6.15 | 66.09 | 4191 | 23.233 | 4.29 | 251 |
| Dino_bin8 | 97.50 | 1.00 | 65.64 | 3299 | 42.57 | 3.54 | 162 |
| Dino_bin9 | 98.35 | 3.30 | 64.38 | 4734 | 24.35 | 5.02 | 478 |
| Dino_bin11 | 95.30 | 2.85 | 65.61 | 4027 | 38.79 | 3.81 | 639 |
| Dino_bin12 | 95.19 | 2.41 | 69.75 | 5849 | 43.16 | 5.79 | 168 |
| Dino_bin13 | 93.35 | 1.42 | 68.22 | 4553 | 21.05 | 4.73 | 297 |
| Dino_bin14 | 99.04 | 0.53 | 70.83 | 6387 | 43.88 | 6.61 | 225 |
| Dino_bin15 | 91.25 | 0.99 | 69.82 | 3595 | 92.23 | 3.55 | 71 |
| Dino_bin16 | 97.44 | 0.00 | 64.35 | 3909 | 34.50 | 3.39 | 158 |
| Dino_bin17 | 92.17 | 1.64 | 72.19 | 4111 | 18.34 | 4.09 | 610 |
| Dino_bin19 | 91.86 | 1.94 | 33.53 | 3324 | 11.61 | 3.06 | 396 |
| Dino_bin20 | 92.80 | 3.47 | 70.82 | 5457 | 43.05 | 5.43 | 733 |
| Dino_bin21 | 97.68 | 1.65 | 67.94 | 5848 | 69.43 | 6.10 | 139 |
| Dino_bin22 | 99.74 | 0.74 | 70.34 | 6329 | 61.99 | 6.15 | 169 |
| Dino_bin23 | 92.57 | 3.86 | 70.35 | 4455 | 13.11 | 4.52 | 483 |
| Dino_bin24 | 96.58 | 4.56 | 69.63 | 3610 | 12.72 | 3.65 | 511 |
| Dino_bin25 | 92.59 | 2.38 | 70.94 | 8249 | 41.40 | 8.35 | 284 |
| Dino_bin26 | 90.40 | 6.36 | 50.74 | 3867 | 20.86 | 3.21 | 169 |
| Dino_bin29 | 91.11 | 3.23 | 54.19 | 4862 | 25.97 | 4.23 | 211 |
| Dino_bin30 | 98.77 | 4.70 | 68.74 | 3401 | 15.32 | 3.34 | 318 |
| Dino_bin31 | 95.07 | 2.66 | 70.23 | 4263 | 57.94 | 4.00 | 121 |
| Dino_bin32 | 97.86 | 0.85 | 64.10 | 3771 | 18.25 | 3.59 | 485 |
| Dino_bin33 | 98.65 | 0.81 | 68.90 | 3929 | 40.93 | 4.58 | 182 |
| Dino_bin34 | 93.23 | 0.42 | 63.76 | 2957 | 82.83 | 3.53 | 73 |
| Dino_bin35 | 96.30 | 3.28 | 61.65 | 3952 | 11.01 | 3.75 | 448 |
| Dino_bin37 | 94.15 | 2.22 | 64.84 | 5059 | 90.20 | 5.22 | 95 |
| Dino_bin38 | 93.04 | 0.09 | 56.96 | 2623 | 27.59 | 2.35 | 109 |
| Dino_bin39 | 90.48 | 3.96 | 65.58 | 2741 | 16.16 | 2.96 | 269 |
| Dino_bin40 | 95.84 | 0.95 | 63.41 | 5560 | 38.50 | 5.85 | 235 |
| Dino_bin41 | 96.63 | 2.31 | 72.88 | 4695 | 39.29 | 4.96 | 217 |
| Dino_bin42 | 97.86 | 0.85 | 65.53 | 3656 | 27.56 | 3.52 | 343 |
| Dino_bin43 | 98.40 | 1.49 | 65.10 | 5848 | 46.19 | 6.76 | 242 |
| Dino_bin44 | 96.58 | 3.42 | 64.52 | 5879 | 60.66 | 6.78 | 382 |
| Dino_bin45 | 98.06 | 2.91 | 38.71 | 4761 | 44.56 | 3.89 | 380 |
| Dino_bin46 | 94.20 | 1.52 | 67.36 | 4186 | 37.58 | 4.42 | 317 |
| Dino_bin47 | 90.51 | 1.76 | 66.18 | 9241 | 44.87 | 7.35 | 208 |
| Dino_bin48 | 98.28 | 7.52 | 72.91 | 5248 | 14.20 | 5.34 | 494 |
| Dino_bin49 | 96.36 | 2.35 | 69.01 | 4957 | 38.43 | 5.27 | 188 |
| Dino_bin50 | 90.46 | 5.13 | 64.38 | 6606 | 82.78 | 6.52 | 97 |
| Dino_bin54 | 94.78 | 2.71 | 65.93 | 4327 | 280.43 | 4.55 | 36 |
| Dino_bin55 | 96.51 | 1.85 | 70.14 | 4581 | 43.91 | 4.69 | 622 |
| Dino_bin56 | 94.59 | 1.28 | 65.03 | 3367 | 13.25 | 2.25 | 227 |
| Dino_bin57 | 94.33 | 1.95 | 68.96 | 4947 | 25.99 | 5.10 | 688 |
| Dino_bin58 | 95.47 | 3.26 | 68.28 | 4118 | 39.37 | 4.36 | 373 |

**Table S2** Statistical summary for MAGs recovered from microbiome in dinosaur fossil bone.
