# Supplementary material for: Genome-centric resolution of novel microbial lineages in an excavated Centrosaurus dinosaur fossil bone from the Late Cretaceous of North America
Source: Environ Microbiome. 2020 Mar 19;15:8. doi: 10.1186/s40793-020-00355-w (PMC8067395; doi:10.1186/s40793-020-00355-w)
Supplement: Supplementary file 6 — Additional file 6: Table S5 Taxonomic identification of MAGs based on homology of 16S rRNA gene. [file 40793_2020_355_MOESM6_ESM.docx]

**Table S5** Taxonomic identification of MAGs based on homology of 16S rRNA gene.

| MAGs | Length (bp) | Best hit of cultured organisms (identity) | Best hit of uncultured organisms (identity, %) | Taxonomic group (phylum or class level) |
| --- | --- | --- | --- | --- |
| Dino_bin26 | 1538 | Nitrospira marina Nb-295 (98%) | Uncultured bacterium clone CT0C1AB06 (99%) | Nitrospira |
| Dino_bin29 | 1555 | Desulfomonile tiedjei DSM 6799 (87%) | Uncultured bacterium clone F1_44X2 (99%) | Deltaproteobacteria |
| Dino_bin7 | 416 | Vicinamibacter silvestris strain Ac_5_C6 (90%) | Uncultured bacterium 1112842459969b (99%) | Acidobacteria |
| Dino_bin37 | 1475 | Bradyrhizobium sp. Shinshu-th2 (95%) | Uncultured bacterium clone 3BR-4E (99%) | Alphaproteobacteria |
| Dino_bin43 | 1529 | Burkholderiales bacterium GJ-E10 (93%) | Uncultured bacterium clone SPN400-300day-33 (99%) | Betaproteobacteria |
| Dino_bin34 | 1187 | Dehalogenimonas lykanthroporepellens BL-DC-9 (84%) | Uncultured bacterium clone SIFF406_N9D4 (98%) | Chloroflexi |
| Dino_bin38 | 1490 | Dehalogenimonas lykanthroporepellens strain BL-DC-9 (83%) | Uncultured bacterium clone S1_86 (96%) | Chloroflexi |
| Dino_bin39 | 1495 | Caldilinea aerophila DSM 14535 (85%) | Uncultured bacterium clone ncd2413g07c1 (95%) | Chloroflexi |
| Dino_bin11 | 407 | Jatrophihabitans endophyticus strain S9-650 (86%) | Uncultured Actinomycetales bacterium clone CNY_01369 (93%) | Actinobacteria |
| Dino_bin12 | 588 | Actinomadura geliboluensis strain XY227 (93%) | Uncultured actinobacterium clone BWET1cm36 (94%) | Actinobacteria |
| Dino_bin16 | 1494 | Sporichthya polymorpha strain AL42 (87%) | Uncultured bacterium clone F1_59X_2 (98%) | Actinobacteria |
| Dino_bin24 | 1476 | Euzebya sp. DY32-46 (92%) | Uncultured bacterium clone AC14C2CH04 (95%) | Actinobacteria |
| Dino_bin32 | 1381 | Allostreptomyces psammosilenae strain YIM DR4008 (87%) | Uncultured bacterium clone 4-262 (94%) | Actinobacteria |
| Dino_bin33 | 1504 | Kribbella flavida DSM 17836 (94%) | Uncultured bacterium clone 11-752 (96%) | Actinobacteria |
| Dino_bin48 | 1379 | Aciditerrimonas ferrireducens strain IC-180 (92%) | Uncultured bacterium clone HDB_SIOO1071 (98%) | Actinobacteria |
